# The Isolated in Utero Environment Is Conducive to the Emergence of RNA and DNA Virus Variants

**DOI:** 10.3390/v13091827

**Published:** 2021-09-14

**Authors:** Daniel Udenze, Ivan Trus, Henry Munyanduki, Nathalie Berube, Uladzimir Karniychuk

**Affiliations:** 1 Vaccine and Infectious Disease Organization (VIDO), University of Saskatchewan, Saskatoon, SK S7N 5E3, Canada; dou286@mail.usask.ca (D.U.); ivan.trus@gmail.com (I.T.); Henry.Munyanduki@pirbright.ac.uk (H.M.); nab602@mail.usask.ca (N.B.); 2 School of Public Health, University of Saskatchewan, Saskatoon, SK S7N 2Z4, Canada; 3 Department of Veterinary Microbiology, Western College of Veterinary Medicine, University of Saskatchewan, Saskatoon, SK S7N 5B4, Canada

**Keywords:** viral evolution, intra-host evolution, Zika virus, porcine circovirus, fetus, placenta, pregnancy

## Abstract

The host’s immune status may affect virus evolution. Little is known about how developing fetal and placental immune milieus affect virus heterogeneity. This knowledge will help us better understand intra-host virus evolution and how new virus variants emerge. The goal of our study was to find out whether the isolated in utero environment—an environment with specialized placental immunity and developing fetal immunity—supports the emergence of RNA and DNA virus variants. We used well-established porcine models for isolated Zika virus (RNA virus) and porcine circovirus 2 (DNA virus) fetal infections. We found that the isolated in utero environment was conducive to the emergence of RNA and DNA virus variants. Next-generation sequencing of nearly whole virus genomes and validated bioinformatics pipelines identified both unique and convergent single nucleotide variations in virus genomes isolated from different fetuses. Zika virus and PCV2 in utero evolution also resulted in single nucleotide variations previously reported in the human and porcine field samples. These findings should encourage further studies on virus evolution in placenta and fetuses, to better understand how virus variants emerge and how in utero viral evolution affects congenital virus transmission and pathogenicity.

## 1. Introduction

The emerging concept is that pregnancy-modulated maternal immunity is favorable for virus evolution towards a more pathogenic infection phenotype, i.e., a failure to mount an efficient antiviral response in pregnant mice is associated with the emergence of influenza variants with increased pathogenicity [1]. Along with the pregnancy-modulated maternal immunity, the developing fetal and placental immune milieus may potentially impose different pressures on viral evolution. However, little is known about viral evolution in fetuses and placenta.

Limitations imposed by the sampling of human tissues restrict comprehensive studies on virus evolution in fetuses. Thus, viruses that cause in utero infections in animals may serve as valuable models to study the complexity of viral evolution in fetuses. However, only a few research efforts on in utero viral evolution in the natural animal host are reported [2,3,4]. The common for previous animal model studies is that most of them were conducted with partial virus genome sequencing with Sanger technology. Thus, the next-generation sequencing (NGS) of whole-virus genomes and sensitive mutant spectrum complexity analysis was not applied. Additionally, the experimental designs of previous studies with both maternal and in utero infections did not allow for distinguishing whether new virus variants emerged in fetuses or in mothers.

Viral evolution studies in fetuses may increase our fundamental understanding of intra-host virus heterogeneity and virus variant emergence. Towards this goal, we designed a focused study aiming to understand whether an isolated in utero environment—the environment with the developing fetal and placental immunity—is conducive to the emergence of RNA and DNA virus variants. We used well-established porcine models for isolated in utero ZIKV and porcine circovirus 2 (PCV2) infections. Afterward, using the NGS of nearly whole-virus genomes and validated bioinformatics pipelines, we profiled the in utero heterogeneity of ZIKV and PCV2 genomes.

## 2. Materials and Methods

### 2.1. Generation of RNA and DNA Virus Stocks

To reduce virus genomic heterogeneity and reproduce a hypothetical scenario wherein founder viruses are transmitted via the placental barrier [5], we generated ZIKV and PCV2 stocks with reverse genetics and infectious clones. For RNA virus, we used the Infectious Subgenomic Amplicon (ISA)-derived Asian ZIKV H/PF/2013 strain (GenBank: KJ776791.2) [6]. Three overlapping DNA fragments covering the whole ZIKV genome (at positions 1–3428, 3354–7621, and 7553–10,807 nucleotides) were de novo synthesized (GenScript) and inserted into the pUC57 plasmids. Fragments were amplified with the Platinum™ PCR SuperMix High Fidelity PCR kit [6] (Thermo Fisher Scientific, Waltham, MA, USA), transfected into C6/36 Aedes albopictus mosquito cells (ATCC; CRL-1660) at +37 °C for 12 h with Lipofectamine^®^ 3000 (Thermo Fisher Scientific), and incubated for 7 days at +28 °C. After two passages in C6/36 cells, cell culture media containing ZIKV was centrifuged (12,000× *g*, 20 min, +4 °C) and frozen (−80 °C). Viral titers were quantified in triplicates in VERO cells (ATCC; CRL-1586) with an endpoint dilution assay, as described below.

For the DNA virus, the whole genome of the PCV2 strain BaPCV2b (GenBank: FJ233905.1) was de novo synthesized (GenScript). The circular BaPCV2b genome has a single SacII restriction site at 491–496 nt; the DNA was synthesized starting from 495 nt with CGGC and GG added to the 5’ and 3´ fragment ends to generate two SacII flanking restriction sites (File S1). The extended DNA fragment was inserted into the pUC57 plasmid at the multiple cloning site (EcoRV). The plasmid was amplified with the isothermal rolling-circle method using the TempliPhi 100 Amplification kit (GE Healthcare) and digested with SacII restriction enzyme (New England Biolabs, Ipswich, MA, USA); the 1767 bp band was selected with preparative gel electrophoresis, purified with an Invitrogen PureLink Quick Gel Extraction Kit (Thermo Fisher Scientific, Waltham, MA, USA), circularized with T4 DNA Ligase (New England Biolabs, Ipswich, MA, USA), and transfected into VR1BL cells [7] with Lipofectamine^®^ 3000 (Thermo Fisher Scientific, Waltham, MA, USA). To generate sufficient titers for the in vivo infection study, supernatants were passaged three times in VR1BL and three times in Dulac cells [8]; cell culture media containing PCV2 was centrifuged (12,000× *g*, 20 min, +4 °C) and frozen (−80 °C). Viral titers were quantified in Dulac cells with the endpoint dilution assay as described below.

The absence of mycoplasma contamination was confirmed in all virus stocks and cell cultures with the LookOut Mycoplasma PCR Detection Kit (Sigma-Aldrich, St. Louis, MO, USA).

### 2.2. Animal Experiments

We followed the Canadian Council on Animal Care guidelines (Animal Use Protocol approval #20180012 from the University of Saskatchewan’s Animal Research Ethics Board). All efforts were made to minimize animal suffering. Pigs were humanely euthanized with Euthanyl overdose followed by exsanguination. We used well-established porcine models for isolated ZIKV [9,10,11,12,13,14] and PCV2 [15,16] in utero infection. Two conventional time-pregnant Landrace-cross pigs were purchased from a high-health status herd free from porcine reproductive and respiratory syndrome virus (PRRSV) and porcine parvovirus (PPV), which can cause fetal infections in pigs, and congenital PCV2 and PCV3 infections. In addition, fetal samples were tested by PCR for PRRSV, PPV, and PCV3 after sampling, and confirmed to be negative. Animals were housed at Vaccine and Infectious Disease Organization biosafety level 2 facilities. One of the pregnant pigs was in utero inoculated with ZIKV at 50 gestation days (gd) [9,10,12,13,14]; another pig was inoculated with PCV2 at 50 gd [17,18] (the total duration of porcine pregnancy is 114–115 days). For precise inoculation, we used an ultrasound-guided technique, which allows for verifying fetal viability before and after injection by visualizing the heart beating [9,10,12,13,14]. We inoculated four conceptuses (a fetus with fetal membranes) with ZIKV and five conceptuses with PCV2. Each conceptus was inoculated intraperitoneally + intra-amniotic (100 μL + 100 μL) with 2 × 10^5^ TCID_50_ of ZIKV or intraperitoneally + intra-amniotic (200 μL + 500 μL) with 10^3.9^ TCID_50_ of PCV2. Conceptuses were labeled with non-absorbable surgical sutures on the adjacent uterine walls to directly identify inoculated fetuses during sampling. Pigs were sampled 28 days after ZIKV and PCV2 in utero inoculation [9,10,12,13,14]. Uteri with fetuses were removed, and all samplings were performed from the most distant non-manipulated conceptus to the inoculated fetuses, with dedicated instruments for each fetus. In both ZIKV and PCV2 pigs, a uterine wall with the placenta (fetal placental compartment was subsequently dissected from the maternal endometrium) was collected from each conceptus and rapidly frozen. Umbilical cord blood was aspirated from each fetus with sterile syringes and needles before fetal tissue dissection. After blood centrifugation (2000× *g*, 20 min, +4 °C), plasma was aliquoted and frozen (−80 °C). Fetuses were visually examined, and gross pathology was recorded. Fetal organs were sampled based on known ZIKV [9,10,12,13,14] and PCV2 [17,18] tissue tropism: For ZIKV, fetal brains and inguinal lymph nodes were collected and frozen in liquid nitrogen. For PCV2, the fetal heart, spleen, and brains were collected and frozen in liquid nitrogen. Virus loads and virus genomic heterogeneity were tested in tissues as described below.

### 2.3. Quantification of ZIKV Loads and Virus-Specific Antibodies

We used the endpoint dilution assay to quantify infectious titers in the ZIKV stock [9,10,12,13,14]. The virus stock was serially diluted five-fold in four replicates starting from 1:5 in DMEM media (Thermo Fisher Scientific, Waltham, MA, USA) supplemented with 5% fetal bovine serum (FBS) (Sigma-Aldrich, St. Louis, MO, USA), a mixture of antibiotics (1000 IU/mL penicillin and 1 mg/mL streptomycin, Gibco, Amarillo, TX, USA), and 2.25 g/L sodium bicarbonate (Thermo Fisher Scientific, Waltham, MA, USA). In total, 50 μL of each dilution was added to confluent VERO cells cultured in 96-well plates. After 2 h of incubation, 150 μL of fresh media was added to each well. The cells were incubated for 7 days. After washing and drying, the plates were kept at −20 °C for at least 2 h or until use. Anti-pan flavivirus E protein monoclonal antibodies 4G2 (ATCC; HB-112) were used to detect ZIKV-infected cells [9,10,12,13,14]. Then, 50% endpoint titers were calculated by the Spearman–Kärber formula and expressed in a decimal logarithm of a 50% infection dose for cell cultures (log10 TCID_50_) per ml. Media from mock-inoculated cells were used as negative controls.

Despite the presence of infectious virus in amniotic fluids, as well as persistent infection and molecular pathology in fetal organs, the isolation of infectious ZIKV from tissues of immunocompetent animals is challenging, and the reverse transcriptase quantitative polymerase chain reaction (RT-qPCR) assay is a generally used approach to determine in vivo viral loads [14]. To confirm in utero infection and quantify ZIKV genomic RNA copies for NGS library construction, fetal placenta, brain, and lymph nodes were dissected and weighed on analytical balances. For brain tissues, 1 ml of TRI Reagent Solution (Thermo Fisher Scientific, Waltham, MA, USA) was added to 20–40 mg tissues prior to homogenization (5 min at 25 Hz) with RNase-free stainless-steel beads and TissueLyser II (QIAGEN, Hilden, Germany). Then, RNA extraction was performed with PhaseMaker tubes (Thermo Fisher Scientific, Waltham, MA, USA) and a PureLink RNA Mini Kit (Thermo Fisher Scientific, Waltham, MA, USA) according to the manufacturer’s instructions. RNA extractions from homogenized placental, fetal and maternal lymph node tissues were performed with a PureLink RNA Mini Kit according to the manufacturer’s instructions. PCR reactions were conducted on the StepOne Plus platform (Applied Biosystems, Foster City, CA, USA) and analyzed using StepOne 2.3 software. ZIKV-specific SYBRgreen-based one-step RT-qPCR was used for ZIKV RNA quantification [19]. The reaction mixture (20 μL) consisted of 10 μL 2× SensiFAST SYBR Hi-ROX One-Step Mix (Bioline; BIO-73005), 0.4 μL RiboSafe RNase Inhibitor, 0.2 μL reverse transcriptase, 0.8 μL (400 nM) of each primer (ZIKV-F10287: 5′-AGGATCATAGGTGATGAAGAAAAGT-3′; ZIKV-R10402: 5′-CCTGACAACACTAAGATTGGTGC-3′), 3.8 μL nuclease-free water and 4 μL RNA template. A reverse transcription step of 10 min at +45 °C and an enzyme activation step of 2 min at +95 °C were followed by 40 amplification cycles (5 s at +95 °C and 34 s at +60 °C). A standard curve [6] was used to quantify viral ZIKV RNA loads. PCR values were corrected for tissue weights and upon log transformation expressed as ZIKV RNA genome copies per gram. Strict precautions were taken to prevent PCR contamination. Aerosol-resistant filter pipette tips and disposable gloves were used. Kit reagent controls were included in every RNA isolation and PCR run. In all endpoint dilution assays and PCR tests, we included samples from mock-inoculated and non-manipulated animals from our previous studies [9,10,11,12,13,14] as controls. Zika virus-specific antibodies in maternal blood were determined with an immunoperoxidase monolayer assay (IPMA) [9,10,11,12,13,14].

### 2.4. Quantification of PCV2 Loads and Virus-Specific Antibodies

The endpoint dilution assay is a standard method for quantifying infectious PCV2 titers in stock and confirming in utero infection [17,18]. Tissue samples (fetal heart, spleen, placenta, and brain tissues) were weighed and homogenized in 1000 μL serum-free media as described above to form suspensions. Then, tissue suspensions as well as virus stock and blood plasma were serially four-fold diluted in four replicates starting from undiluted, and 50 μL of each dilution was added to confluent Dulac cells (a kind gift from Dr. Allan) cultured in 96-well plates. Dilutions were made in MEM media supplemented with 5% FBS, a mixture of antibiotics, and 2.25 g/L Sodium Bicarbonate. After 2 h of incubation, 150 μL of fresh media was added to each well. The cells were incubated for 7 days. After washing and drying, the plates were kept at −20 °C for at least 2 h or until use. Fixation and staining were performed, as previously described [6,16,17]. The primary mouse anti-PCV2 monoclonal antibodies (RTI; PCV-2-A) and secondary goat anti-mouse horseradish-peroxidase (HRP)-labeled IgG (Abcam, Cambridge, United Kingdom) were used to detect PCV2-infected cells. Fifty percent endpoint titers were calculated by the Spearman–Kärber formula and expressed in log10 TCID_50_ per ml or gram.

We used qPCR to quantify the PCV2 genomic DNA copies in the virus stock, body fluids, and tissues for NGS library construction. We extracted DNA with the PureLink DNA/RNA Mini Kit (Thermo Fisher Scientific, Waltham, MA, USA) from fetal blood plasma, heart, spleen, placenta, and brain, according to the manufacturer’s instructions. Before DNA extraction, the tissue samples were weighed on an analytical balance. Tissues were homogenized using RNase-free stainless steel beads and TissueLyser II operated for 5 min at 25 Hz. We used PCV2-specific SYBR green-based one-step qPCR 13 with a reaction mixture (50 μL) consisting of 25 μL 2× SensiFAST SYBR Lo-ROX Kit Mix (Bioline; BIO-94005), 2 μL (10 µM) of each primer (PCV2-CF8: 5′-TAGGTTAGGGCTGTGGCCTT-3′; PCV2-CR8: 5′- CCGCACCTTCGGATATACTG-3′), 19 μL nuclease-free water and 2 μL DNA template. The amplification of PCV2 DNA was performed via 35 cycles of denaturing at +95 °C for 15 s, annealing at +65 °C for 15 s, and extension at +72 °C for 10 s. DNA from a stock of BaPCV2b was used to generate a standard curve (882 bp, PCV2-F938: 5′-CCAGTTCGTCACCCTTTCC-3′; PCV2-R52: 5′-ATGTTGCTGCTGAGGTGCT-3′) with a wide dynamic range, with the high linear correlation (R2 = 0.9984) between the cycle threshold (Ct) value and template concentration. The slope of the standard curve (−3.358) corresponded to the 98.52% reaction efficiency level. The PCR values were corrected for fluid volumes or tissue weights and after log transformation expressed as PCV2 DNA genome copies per ml or gram. Kit reagent controls were included in every DNA isolation and PCR run. In all endpoint dilution assays and PCR tests, we included samples from mock-inoculated and non-manipulated animals from our previous studies [9,10,11,12,13,14] as controls. Porcine circovirus 2-specific antibodies in maternal blood were determined with IPMA [17,18].

### 2.5. Next-Generation Sequencing

RNA and DNA from virus stocks and placental and fetal samples were used to construct ZIKV and PCV2 whole-genome NGS libraries. We used the PrimalSeq protocol, an approach wherein a nearly whole virus genome is amplified in the ~400–480 bp overlapping fragments with multiplexed PCR reactions and two technical replicates for each multiplex reaction [20]. The amplicons from multiplex PCR reactions are combined for library preparation and NGS. Zika virus RNA was reverse transcribed into cDNA using ProtoScript^®^ II Reverse Transcriptase (20 μL reactions; New England Biolabs, Ipswich, MA, USA) and Random Hexamers (2 μL; Thermo Fisher Scientific, Waltham, MA, USA). As previously validated for accurate iSNV calling, we used more than 1000 viral RNA copies for cDNA synthesis [20]. Zika virus cDNA (2 μL) was amplified in two multiplexed ZIKV-specific PCR reactions with Primal Scheme primers (Table S1). These two multiplex primer schemes were previously designed with a web-based tool, Primal Scheme, for the ZIKV PRVABC59 strain (GenBank: KU501215.10 [20]. To account for genomic differences between the ZIKV PRVABC59 strain and H/PF/2013 strain used in this study, we modified ZIKA_400_1_LEFT, ZIKA_400_17_RIGHT and ZIKA_400_32_LEFT primers, and added the ZIKA_400_36_LEFT and ZIKA_400_36_RIGHT primer pair (Table S1). Altogether, the primer pairs amplified 410–480 nt products with a nearly 100 nt overlap covering nearly the entire ZIKV genome. Each multiplexed reaction was performed in two technical replicates with Q5^®^ Hot Start High-Fidelity 2× Master Mix (25 μL reactions; New England Biolabs). An enzyme activation step of 30 s at +95 °C was followed by 35 amplification cycles for 15 s at +95 °C and 5 min at +65 °C. The correct size of amplicons in each reaction was verified by 1% agarose gel electrophoresis.

Library construction and Illumina sequencing were performed as previously described with some modifications [20]. Virus amplicons from the two multiplex PCR reactions were combined (separately for each technical cDNA replicate) and purified using magnetic beads (1.8:1 ratio of beads (45 μL) to combined sample (25 μL)) from the Illumina TruSeq Nano DNA High Throughput Library Prep Kit (96 samples). DNA concentrations were measured with the Qubit dsDNA HR Assay Kit and Qubit 3.0 Fluorometer (Thermo Fisher Scientific, Waltham, MA, USA). In total, 50 ng of purified DNA in 60 μL was used for library construction with the TruSeq Nano DNA High Throughput Library Prep Kit and TruSeq DNA CD Indexes (96 Indexes, 96 Samples) according to the manufacturer’s protocol. Individual sample libraries were quantified with the Qubit dsDNA HR Assay Kit and Qubit 3.0 Fluorometer. The expected peak DNA fragment size (~580) was confirmed with the Agilent DNA 1000 kit on Agilent 2100 Bioanalyzer (Agilent). In total, 20 ng of each barcoded library was pooled, quantified (Qubit 3.0), quality-checked (Agilent Bioanalyzer), and converted to moles: Molecular weight [nM] = Library concentration [ng/µL]/((Average library size × 650)/1,000,000). The pooled library was diluted to 2 nM in 10 mM TE, denatured with 0.1 N NaOH and diluted to 14 pM, and paired-end 300 nt reads were generated with MiSeq Reagent kit v3 (600 cycle output; Illumina, San Diego, USA) on the MiSeq System (Illumina, San Diego, CA, USA).

The multiplex primer design tool Primal Scheme has previously been used to sequence RNA viruses—ZIKV, yellow fever virus and West Nile virus [20]. Here, we used Primal Scheme to design six pairs of primers for PCV2 with a circular single-stranded DNA genome (Table S1). A pooled primer set failed to amplify the whole PCV2 genome and did not show correct bands in the gel electrophoresis. Non-pooled individual primer pairs, however, amplified bands with the correct sizes (360–420 bp) and showed the expected Sanger-derived sequences. Thus, the whole PCV2 genome from the virus stock and tissue samples was amplified with six individual PCR reactions. We used 1 µL of sample DNA and Q5^®^ Hot Start High-Fidelity 2× Master Mix (12.5 μL reactions). Each individual reaction was performed in two technical duplicates. An enzyme activation step of 30 s at +98 °C was followed by 35 amplification cycles for 7 s at +98 °C, 25 s at +68 °C, and 30 s at +72 °C. The correct sizes of the amplicons in each reaction were verified by 1% agarose gel electrophoresis. Amplicons from the six individual PCR reactions (for each technical replicate) were combined, bead-purified, and used for library construction and sequencing, as described above for ZIKV.

### 2.6. Illumina Data Processing and Variant Calling Using iVar

For NGS data processing and variant calling, we used an open-source software package iVar (intra-host variant analysis from replicates) to process virus sequencing data and call single nucleotide variations (SNVs) from technical replicates [20]. To identify SNVs, we compared ZIKV sequences from experimental samples to a reference sequence. As a reference for ZIKV stock sequences and all ZIKV sequences from in vivo samples, we used the ancestral H/PF/2013 strain (GenBank: KJ776791.2); synonymous and non-synonymous mutations that were not present in the reference sequence were considered as SNVs. Then, we compared the genomic positions and frequencies of SNVs between sequences from the initial ZIKV stock used for fetal inoculation and sequences from in vivo samples; tissue-specific SNVs that were not present in the initial ZIKV stock were considered as in utero-emerged SNVs (iSNVs). The same approach was used for SNV and iSNV analysis in the PCV2 experiment, but the ancestral BaPCV2b strain (GenBank: FJ233905.1) was used as the reference.

As per the previously described and validated best practices [20], we analyzed virus genomic regions with a sequencing depth of at least 400×. Only SNVs/iSNVs with a frequency of at least 3% detected in both technical replicates were considered for analysis; insertions and deletions were not analyzed. In addition, the genomic regions amplified with primers that contained nucleotide mismatches within the binding sites were omitted. The detailed computational protocol used in this paper for iVar is provided in File S2.

The percentage of SNVs in virus sequences from each sample was calculated by dividing the number of SNV sites by the total number of sequenced nucleotides; the mean percentage from two NGS technical replicates was represented. Mean SNV frequencies were also calculated from NGS technical replicates. To quantify the degree of convergent evolution in ZIKV or PCV2 genomes, any iSNV that emerged in more than one fetus/fetal organ was defined as convergent, and the total convergence was quantified as the percentage of convergent iSNVs from the total number of unique iSNVs.

### 2.7. Statistical Analysis

We used GraphPad PRISM 8. Data are expressed as individual and arithmetic mean values.

## 3. Results

**Figure 1:**
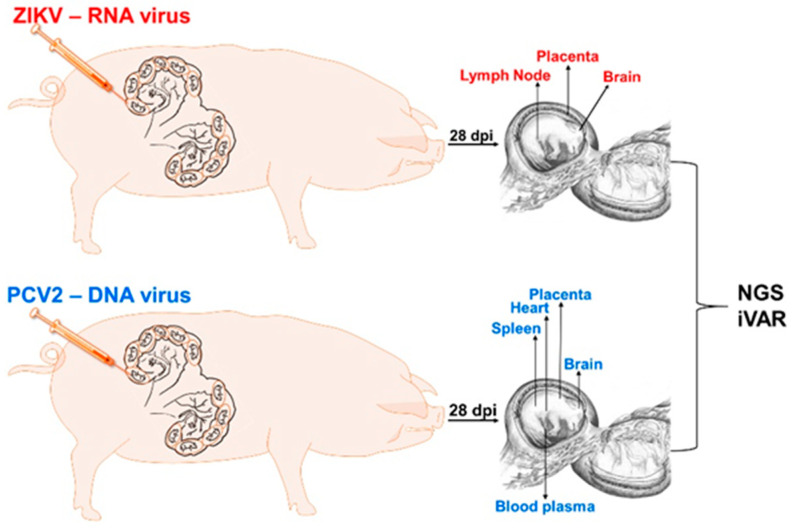
Animal experimental design. dpi: days post-injection. NGS: next-generation sequencing. iVAR: intra-host variant analysis of replicates (github.com/andersen-lab/ivar, accessed on 15 July 2021).

We tested whether the in utero environment is conducive to the emergence of new RNA and DNA virus variants. As a model for RNA viruses, we used ZIKV; as a model for DNA viruses, we used PCV2. The experimental design is shown in Figure 1.

### 3.1. Zika Virus Variants Emerge during In Utero Infection

**Figure 2:**
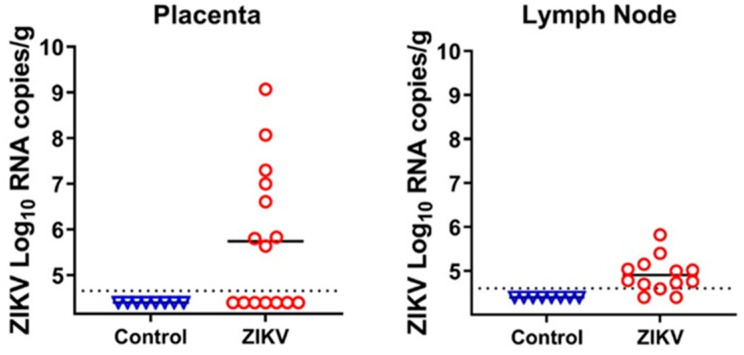
Zika virus loads in the placenta and fetal lymph nodes. Dotted lines represent the limit of detection (LOD), solid lines represent mean values. Samples with PCR Ct values higher than 34 were considered negative [19]. The LOD was determined by calculating the lowest possible virus load at a Ct value of 34, and adding the log10-transformed dilution fold of the tissue sample with the lowest weight. The LOD for placenta—4.7 log10 RNA copies/g; LOD for lymph node—4.6 log10 RNA copies/g. Detailed data are shown in Table S2. Control—tissue samples from healthy virus-negative animals from our previous studies [9,10,11,12,13,14] were used as controls to make sure that reagents were not contaminated.

Fetuses were injected with ZIKV to induce isolated in utero infection. The ultrasound-guided protocol [9,10,11,12,13,14] was used for highly precise fetal injections. In utero inoculation did not cause maternal infection because maternal tissues were negative for ZIKV, and maternal blood did not have ZIKV-specific antibodies. From our previous studies, we know that ZIKV has a specific tropism and persists in the fetal brain and placenta [9,10,11,12,13,14]; in addition to these tissues, we also sampled fetal inguinal lymph nodes. As in previous studies, we found high ZIKV loads in the placental tissues from directly injected and trans-infected fetuses (Figure 2; Table S2). We also found that ZIKV persists in porcine fetal lymph nodes (Figure 2; Table S2). Unexpectedly, the brains of all fetuses were negative for ZIKV (Table S2). While in all our previous studies with the Asian PRVABC59 strain (GenBank: KU501215.1), ZIKV caused infection in fetal brains [9,14], it is possible that the Asian H/PF/2013 strain (GenBank: KJ776791.2) that was used in the present study did not cross the fetal blood–brain barrier, or replicated for a shorter time in the fetal brain. Accordingly, the H/PF/2013 ZIKV strain caused less disseminated in utero infection than PRVABC59 ZIKV in baboons [21]. Thus, we used placental and lymph node tissues to analyze iSNVs in the ZIKV genome.

We extracted RNA from the initial ZIKV stock that was used for fetal injection, placental tissues, and fetal lymph node tissues (Table S2). The extracted RNA was quantified and reverse transcribed, and cDNA was amplified with ZIKV-specific multiplex primers (Table S1) designed with the Primal Scheme tool [20]. As previously validated for accurate SNV calling in the ZIKV genome, we used at least 1000 viral RNA copies for cDNA synthesis [20], except for lymph node samples from two fetuses, where the virus RNA loads were lower (Figure 2; Table S3). Amplified DNA spanning nearly the whole ZIKV genome was used for library constriction, NGS, and SNV analysis.

The initial ZIKV stock and placental samples had correct DNA bands after amplification with multiplex PCR primers, 86.6–98.2% of ZIKV genome coverage, and high (12,437×) average sequencing depth (Figure S1). Out of six fetal lymph nodes, four did not have DNA products and were excluded from library construction (Table S2). Lymph nodes from two fetuses ZIKV-F11-LN and ZIKV-F12-LN had correct ZIKV PCR bands; However, only the ZIKV-F11-LN sample had high (88.8%) coverage of the ZIKV genome (Table S3). Thus, for the analysis of in utero ZIKV heterogeneity, we used data from the placental tissues of five fetuses and the lymph node of one fetus (Table S3).

**Figure 3:**
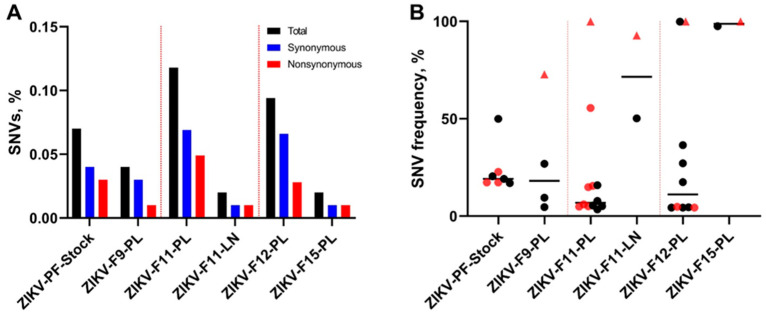
The percentage and frequency of ZIKV SNVs. (**A**) The mean percentage (from two technical replicates) of ZIKV genomic SNVs in virus stock and animal samples calculated from the total number of sequenced nucleotides in each sample. (**B**) The mean frequency (from two technical replicates) of ZIKV genomic SNVs in virus stock and animal samples. Black circles—synonymous SNVs. Red circles—non-synonymous SNVs. Red triangles—the same non-synonymous A3282G SNV identified in samples from different fetuses. Solid lines represent mean values. Raw data are shown in Table S4.

We compared sequences from experimental samples with the parental H/PF/2013 strain sequence (GenBank: KJ776791.2) to identify ZIKV SNVs. We used iVar for NGS processing and variant calling with a sequence depth of at least 400×, a frequency of at least 3%, and variants that were detected in both technical replicates [20]. With these conservative parameters, ZIKV SNVs were identified in the initial virus stock, as well as in the placental and lymph node samples (Table S4). The initial virus stock contained 0.07% of ZIKV genomic SNVs, as calculated from the total number of sequenced nucleotides (Figure 3A). In placental samples, the percentage of SNVs varied from 0.02 to 0.12% (Figure 3A); the placental sample from fetus F8 was excluded from analysis because it had less than 400× depth in one of the replicates (Table S4). The fetal lymph node had 0.02% of the SNVs (Figure 3A). In the initial virus stock, the mean SNV frequencies grouped around 20%, with only one exception of 50% (Figure 3B; Table S4). In placental samples, iSNV frequencies were dispersed from 3.46% to 100% (Figure 3B; Table S4). In fetal lymph node sample, two SNVs had high—50.22% and 92.72%—frequencies (Figure 3B; Table S4).

**Figure 4:**
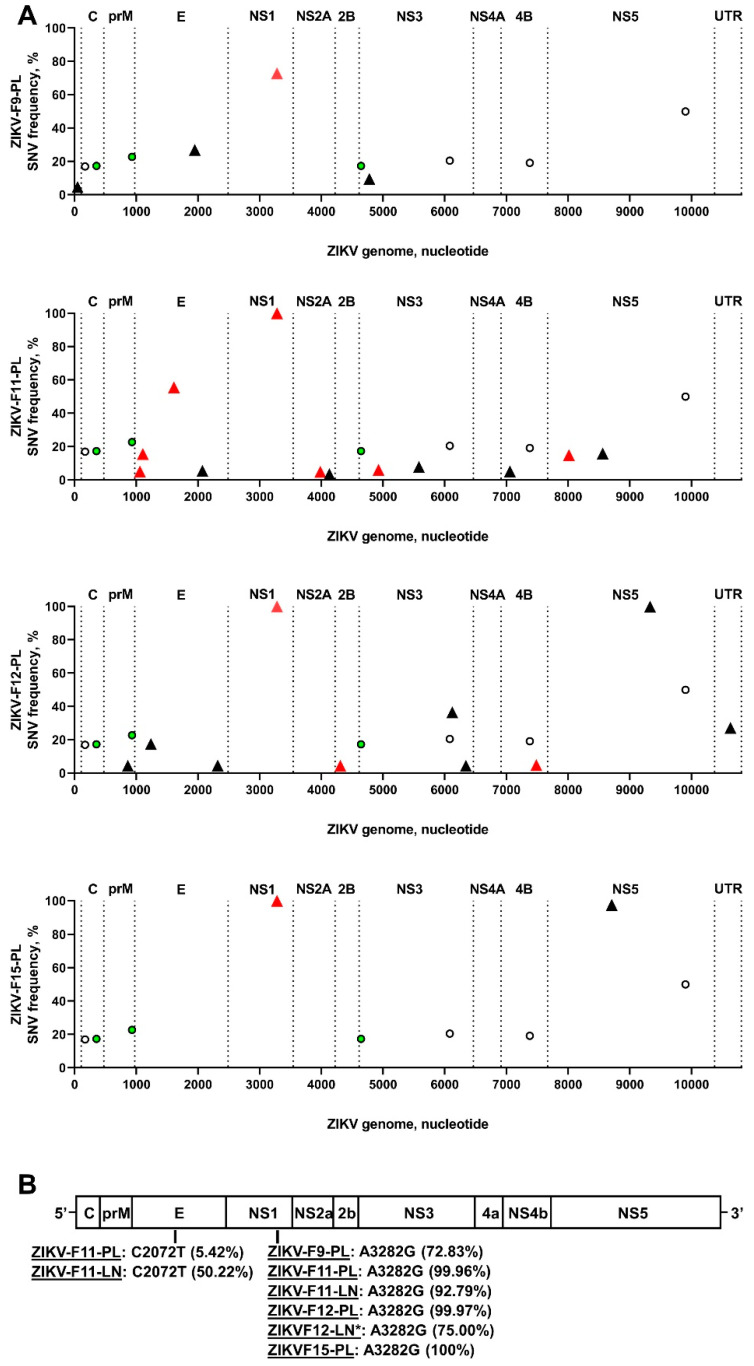
Zika virus iSNVs in placental and fetal tissues. (**A**) Patterns of ZIKV iSNVs in placental samples from individual fetuses. Empty circles—synonymous SNVs in the ZIKV stock. Green circles—non-synonymous SNVs in the ZIKV stock. Black triangles—synonymous ZIKV iSNVs in placental samples. Red triangles—non-synonymous ZIKV iSNVs in placental samples. C: capsid protein. prM: precursor membrane protein. E: envelope protein. NS1: nonstructural protein 1. NS2A: nonstructural protein 2A. NS2B: nonstructural protein 2B. NS3: nonstructural protein 3. NS4B: nonstructural protein 4B. NS5: nonstructural protein 5. (**B**) Convergent iSNVs identified in samples from different fetuses. Raw data are shown in Table S4.

All seven SNVs in the ZIKV stock were most probably introduced during virus rescue with ISA and in vitro virus adaptation to the cell line. While all seven stock-specific SNVs had high frequencies from 17% to 40%, the in utero environment selected against stock-specific SNVs because they were not identified in placental or lymph node samples (Figure 4A; Table S4). To identify iSNVs that emerged in utero, we compared ZIKV sequences from the placenta and lymph node with ZIKV stock sequences; only tissue-specific ZIKV variants that were not present in the initial virus stock were considered as iSNVs. In tissue samples, the number of ZIKV iSNVs varied from 2 to 12 (Figure 4A; Table S4). To confirm that iSNVs are not virus stock-derived artifacts, we called variants in the ZIKV stock sequences with the low frequency threshold (0.01%) and compared low-frequency stock-specific SNVs to tissue-specific iSNVs. This threshold is much below the sequencing error threshold reported for Illumina equipment—0.1–0.46%. In total, 16 tissue-specific iSNVs out of 30 were not identified in the ZIKV stock sequences, even at the 0.01% threshold (Table S4). Thirteen tissue-specific iSNVs were identified in the ZIKV stock sequences; however, the frequencies of these stock SNVs were only 0.01–0.07% (Table S4), that was most probably Illumina equipment artifacts [22,23,24]. One tissue-specific iSNV (A4778G in ZIKV-F9-PL; Table S4) was identified in the ZIKV stock with 0.13% frequency; this stock SNV might be an Illumina equipment error [22,23,24], or an in vitro-acquired SNV that was further selected during infection in the placenta and amplified to 9.44% frequency (Table S4). Additionally, to confirm that virus genome coverage did not affect analysis—i.e., when tissue-specific iSNVs are virus stock-derived artifacts, but coverage gaps in the ZIKV stock sequences influence analysis—we compared NGS coverage gaps in the sequences from virus stock and in vivo samples. Most coverage gaps were identical between sequences from the virus stock and those from in vivo samples (Table S5). The only two uncovered gaps in the virus stock sequences—at the 5308–5483 and 6459–6695 nucleotide positions—did not match well-covered regions in the placental sample from the ZIKV-F12-PL fetus (Table S5); however, these ZIKV genomic regions in the placental sample did not contain iSNVs. Altogether, many placental- and lymph node-specific ZIKV SNVs emerged in utero and were not artifacts of stock-specific SNVs.

Placental and lymph node samples showed unique patterns of ZIKV iSNVs (Figure 4A); however, placental samples from four fetuses and lymph node samples from two fetuses had the same non-synonymous A3282G iSNV with high 73–100% frequencies (Figure 3B and Figure 4B). Another non-synonymous iSNV (C2072T) was identified in both placental and lymph node tissues from the same fetus F11 (Figure 4B). The degree of convergent evolution was 8%. This suggests the independent in utero evolution of similar ZIKV genomic features, and possibly the biological relevance of tissue-specific iSNVs.

We also compared iSNVs to ZIKV sequences available in public databases. Fifteen iSNVs were recently (2014–2020) reported in Asian ZIKV strain sequences from Brazil, Colombia, Dominican Republic, French Polynesia, Honduras, India, Mexico, New Caledonia, Nicaragua, Puerto Rico, Singapore, Suriname, Thailand, and the USA (Table S6). Twelve iSNVs were described in samples from humans and one iSNV from mosquitoes; the origins of two iSNVs were not known. Interestingly, one iSNV (A10633G) was also reported in historical ZIKV strain sequences belonging to an African lineage from Senegal (2001) and Côte d’Ivoire (1999). Altogether, in utero infection resulted in the emergence of virus genomic variants that at least partially resembled natural ZIKV evolution during the 2015 epidemic.

### 3.2. Porcine Circovirus 2 Variants Emerge during In Utero Infection

**Figure 5:**
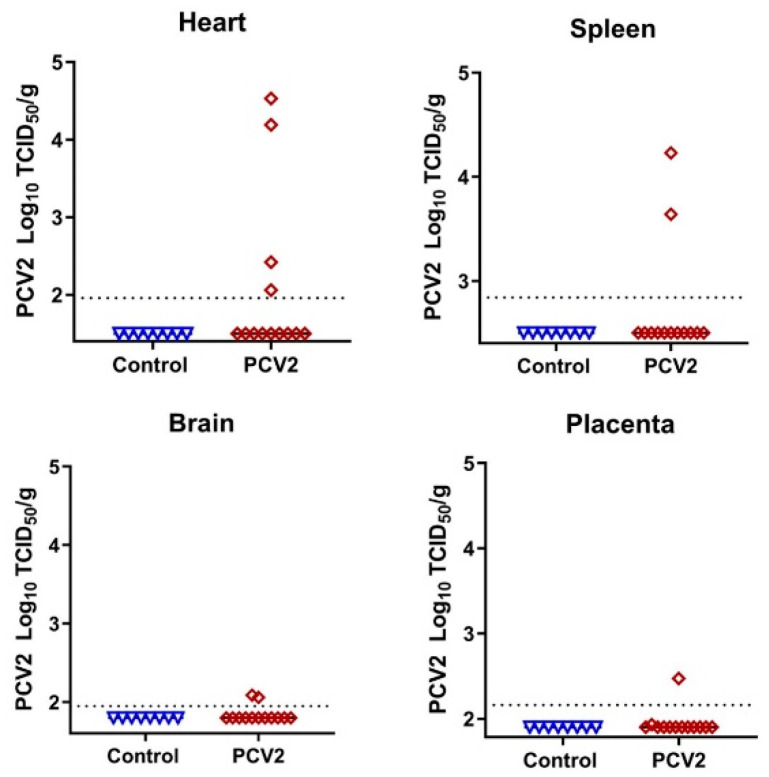
Porcine circovirus 2 loads in fetal organs. Dotted lines represent the LOD, solid lines represent mean values. The LOD was determined by calculating the lowest possible Spearman–Kärber titer under the used titration conditions and adding the log10-transformed dilution fold of the tissue sample with the lowest weight. The LOD for heart—1.96 log10 TCID50/g; the LOD for spleen—2.84; placenta—2.16; brain—1.95. Detailed data are provided in Table S2. Control—tissue samples from healthy virus-negative animals from our previous studies were used as controls to make sure that reagents were not contaminated [9,10,11,12,13,14].

To study whether the in utero environment is conducive to the emergence of new DNA virus variants, we injected five fetuses with PCV2—the virus with the smallest known DNA genome that causes natural infection in pig fetuses. We confirmed high levels of PCV2-specific antibodies in maternal blood before breeding and before in utero inoculation; antibodies prevent maternal infection, thus providing a model for isolated in utero infection. Fetal and placental tissues were sampled 28 days after injection. In utero PCV2 infection caused typical clinical outcomes [15,16]—a mix of normal fetuses, fetuses with edema, hemorrhages, and mummification (Table S2). Infectious PCV2 was identified in the internal organs of directly inoculated fetuses (Figure 5; Table S2); organs from non-manipulated fetuses did not show the infectious virus, which is in agreement with previous studies. The more sensitive quantitative PCR assay confirmed the high virus loads in the blood plasma and organs of all directly injected fetuses (Table S2).

We used DNA extracted from virus stock and from blood plasma, heart, spleen, brain, and placenta tissues from four injected fetuses (tissues from the fifth injected fetus were not available because of mummification; Table S2) to analyze SNVs in the PCV2 genome. High quantities of virus genomic DNA (Table S3) were amplified with PCV2-specific multiplex primers (Table S1) designed with the Primal Scheme tool. Amplified DNA spanning nearly the whole PCV2 genome was used for library construction, NGS, and SNV analysis. The initial PCV2 stock had correct DNA bands after amplification with multiplex PCR primers. In contrast, out of twenty PCV2-positive fetal tissues, eight did not have DNA products, and were excluded from library construction (Table S2). Samples from two fetuses—PCV2-F11-BL and PCV2-F12-SP—were also excluded from SNV analysis because of the low NGS coverage and depth (Table S3; Figure S2). The remaining ten fetal tissues had correct DNA products, 94.4–97.5% PCV2 genome coverage (Table S3), and high (122,101×) average sequencing depth (Figure S2).

**Figure 6:**
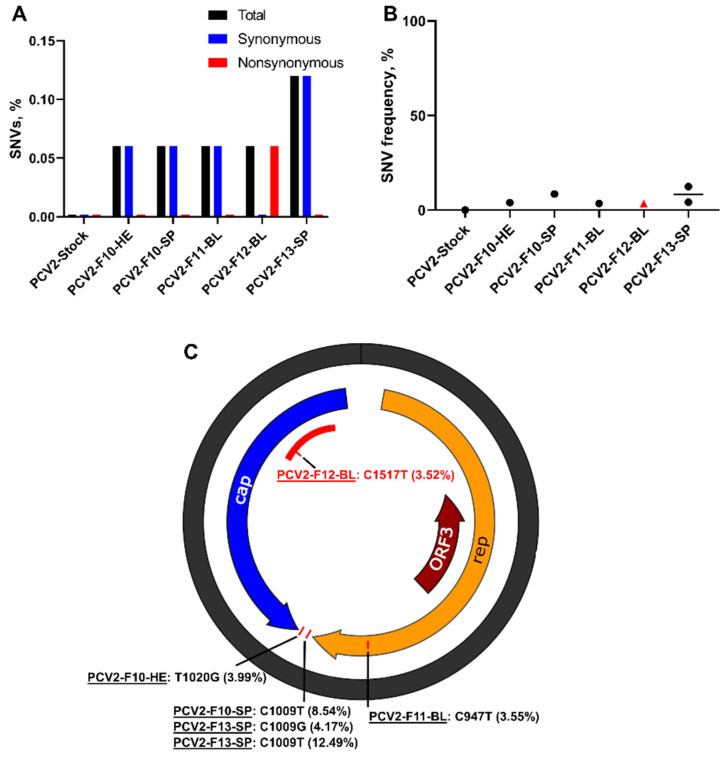
Porcine circovirus 2 iSNVs in placental and fetal tissues. (**A**) The mean percentage (from two technical replicates) of PCV2 genomic SNVs in virus stock and fetal samples calculated from the total number of sequenced nucleotides. (**B**) Mean frequency (from two technical replicates) of PCV2 genomic SNVs in virus stock and animal samples. Black circles—synonymous SNVs. A red triangle—the non-synonymous iSNV (C1517T). Solid lines represent mean values. (**C**) Patterns of PCV2 iSNVs in fetal samples. The same iSNVs (C1009T) were identified in samples from different fetuses. rep: the replication protein encoded by ORF1. cap: the replication protein encoded by ORF2. ORF3: open reading frame 3; the function of the encoded protein is not known. The red area—an antibody recognition domain [25,26]. The non-synonymous iSNV (C1517T) was identified in the antibody recognition domain. Raw data are shown in Table S4.

First, to identify PCV2 SNVs, we compared sequences from stock and animal samples with the ancestral BaPCV2b strain sequence (GenBank: FJ233905.1). Porcine circovirus 2 showed high stability in vitro: SNVs were not identified in the initial virus stock, even after six passages in cell cultures. However, even with high in vitro stability and conservative iVar parameters, de novo-emerging PCV2 iSNVs were identified in six tissues derived from four fetuses (Table S4). The percentage of iSNVs varied from 0.06% to 0.12% (Figure 6A); mean frequencies varied from 3.52% to 12.49% (Figure 6B; Table S4). Synonymous iSNV was identified in the open reading frame 1 (ORF1) encoding the replication protein; the untranslated region also contained two iSNVs (Figure 6C; Table S4). A non-synonymous SNV was identified in the ORF2 encoding the capsid protein; interestingly, this mutation was in the antibody recognition domain [25,26] (Figure 6C). Spleen samples from two fetuses had the same iSNV (C1009T) with 4.2% and 8.5% frequencies (Figure 6C; Table S4). The degree of convergent evolution was 20%, which suggests the independent in utero evolution of similar PCV2 genomic features, and possibly the biological relevance of tissue-specific iSNVs.

To further confirm that tissue-specific iSNVs are not virus stock-derived artifacts, we called variants in the PCV2 stock sequences with a low (0.01%) frequency threshold and compared low-frequency stock-specific SNVs to iSNVs. One iSNV out of six was not identified in the PCV2 stock sequences, even with the very low 0.01% threshold (Table S4). Three tissue-specific iSNVs were identified in the PCV2 stock sequences; however, the frequencies of these stock SNVs were only 0.03–0.2% (Table S4), which are most probably Illumina equipment artifacts [22,23,24]. One iSNV (C1009T in PCV2-F10-SP and PCV2-F13-SP; Table S4) was identified in the PCV2 stock with 1.25% frequency; this stock SNV is probably an in vitro-acquired mutation that was further selected during infection in the fetal spleen and amplified to 8.5–12.5% frequency (Table S4). Virus stock sequences and sequences from in vivo samples selected for SNV analysis did not have NGS coverage gaps that could affect SNV analysis (Figure S2). Altogether, these analyses suggest that at least some PCV2 iSNVs in fetal tissues emerged in utero, and some low-frequency in vitro-acquired SNVs were selected and amplified during infection in fetuses.

We also compared tissue-specific iSNVs to the PCV2 sequences available in public databases. Four PCV2 iSNVs were previously reported in sequences from pig farms in China, Europe, India, Indonesia, and the USA (Table S6). Altogether, in utero infection resulted in the emergence of virus genomic variants that may have biological relevance and resemble natural PCV2 evolution in pigs on farms.

## 4. Discussion

We designed this study to understand whether the isolated in utero environment—a fetus with fetal placental mesenchyme—affects the evolution of RNA (ZIKV) and DNA (PCV2) viruses. The key finding is that the isolated in utero environment is conducive to the emergence of RNA and DNA virus variants. The emergence of these variants can result from the de novo development of mutations, and from the selection of and increase in initial low-frequency variants.

To induce isolated infection in the fetus with fetal placental membranes, we used ISA-derived ZIKV and precise ultrasound-guided in utero inoculation. In utero inoculation did not cause maternal infection because maternal tissues were negative for ZIKV, and the blood did not have ZIKV-specific antibodies. Thus, in our fetal pig model, isolated in utero virus evolution can be analyzed in the context of specific fetal immune responses. Additionally, the fetal pig model reproduces in utero ZIKV infection in humans well; e.g., the virus persists in the fetal placental mesenchyme for at least 90 days [10]. The fetal placental mesenchyme in pigs has all cell types found in the fetal side of the human placenta (chorionic plate), and performs the same fundamental functions. Accordingly, we found that, similar to human placental infection [27], ZIKV infection in the porcine placental mesenchyme is associated with an increased number of CD163-positive cells [9]. Thus, comparable local immune responses to infection in humans and pigs provide a model for comparative virus evolution studies in the fetal placenta. Humans and pigs also show comparable systemic immunity and fetal development [28,29], favoring comparative virus evolution studies in fetal organs.

Each placental sample had a unique pattern of ZIKV iSNVs (Figure 4A), suggesting individual evolutionary pressure in sibling fetuses from the same uterus. While we did not normalize the RNA input for NGS in samples with high ZIKV loads to the sample with the lowest virus load (ZIKV-F8-PL—2597 copies per reaction, which is well above the validated 1000 RNA copies [20], Table S3), several observations support the unique evolutionary pressure evoked by sibling fetuses: Placental samples with a similar ZIKV RNA input (ZIKV-F9-PL-a and ZIKV-F15-PL, Table S3) had different iSNV patterns (Figure 4A). A mouse model for ZIKV evolution has also shown that the inoculation route and virus RNA input for NGS do not affect viral diversity [30]. In addition, cells from human siblings have different susceptibilities to ZIKV [31], and human fetal siblings can manifest different outcomes from congenital ZIKV infection [32]. All the above suggests the unique pressure evoked by sibling fetuses on ZIKV evolution, and this should be taken into account in future studies on how new congenital virus variants emerge. Along with the unique pattern of ZIKV evolution in fetuses, we described convergent evolution as represented by the emergence of identical iSNVs with high frequencies—72.8–100%—in different fetuses (Figure 3B and Figure 4B). Additional studies are necessary to identify whether fetus-specific iSNVs have biological significance and affect ZIKV pathogenicity.

Interestingly, a recent study showed ZIKV circulation in mosquitoes from pig farms in Mexico, as determined by PCR, and natural ZIKV infection in farm pigs, as determined by serological tests [33]. Thus, in addition to the better fundamental understanding of intra-host virus evolution in the fetus, ZIKV studies in pigs may provide new knowledge of the emergence of ZIKV variants in the natural zoonotic reservoir.

Zika virus iSNVs identified in our study were previously reported in human and mosquito samples collected between 2014 and 2020 (Table S6). Thus, in utero infection in the porcine model resulted in the emergence of virus genomic variants that partially resembled natural ZIKV evolution during the 2015 epidemic. This also indirectly suggests that at least some of the ZIKV variants that caused human infections during the 2015 epidemic have initially originated in utero—in infected pregnant women or pregnant zoonotic hosts.

Porcine circovirus 2 is a ubiquitous pig pathogen that naturally causes infection in adults and fetuses. In our PCV2 model, the virus is injected directly into fetuses and does not cause maternal infection, because most adult pigs have immunity against PCV2 that is acquired naturally during the life span or after vaccination [15,16]. In this study, we have also confirmed high levels of PCV2-specific antibodies in maternal blood before breeding and before in utero infection. Beneficially, maternal antibodies do not cross through the porcine placenta and do not affect the isolated in utero environment [34].

We used PCV2 with a low mutant spectrum complexity and precise ultrasound-guided in utero inoculation to induce isolated infection in fetuses. For replication, PCV2 uses the host’s polymerase with proofreading activity, but despite proofreading the polymerase-guided replication, we identified unique PCV2 iSNVs as well as convergent iSNVs in different fetuses (Table S4). In contrast, six passages in cell lines did not increase PCV2 mutant spectrum complexity. As per our findings in fetuses, PCV2 genomic diversity and the association of clinical signs with virus diversity have been described in young pigs [35]. Interestingly, all iSNVs that emerged in fetuses in our study were previously reported in young or adult pigs on farms (Table S6), suggesting that in utero-emerging PCV2 variants may reach susceptible populations outside the maternal body, potentially contributing to the heterogeneity of circulating viruses and outbreaks. This new understanding will be important in future studies on circovirus evolution and viral spreading in pig populations, and potentially in other species, because a thousand highly diverse circular DNA viruses, including circoviruses, have been recently identified in humans and other animals [36].

Porcine and bovine offspring exposed during the fetal period to congenital viruses—classical swine fever virus, Japanese encephalitis virus, and bovine viral diarrhea virus—may shed virus into the environment, and develop disease [2,37,38,39]. Thus, intraspecies or interspecies spillovers of virus variants that emerged in fetuses may occur via offspring that have been infected in utero. To prove this hypothesis, molecular epidemiology studies with rigorous sampling during farm and field outbreaks of congenital infections are necessary to identify whether emerging placental/fetal virus variants spread to older populations, and whether this hypothetical scenario applies for different congenital viruses and in different animal species.

Collectively, we have shown that the isolated in utero environment is conducive to the emergence of RNA and DNA virus variants. These findings should encourage further studies on virus evolution in fetuses and placenta, in order to better understand how congenital virus variants emerge and spread. Additional studies are also urged to identify whether in utero-emerging virus variants may pose more severe transmission and disease development risks. Understanding how in utero evolution affects the transmission and pathogenicity of new virus variants may promote scientific and public health efforts to develop monitoring strategies and preventive interventions, including efficient vaccines that will be safe for pregnant individuals.

## Data Availability

Access to sequencing data files generated in this study is available in the Sequence Read Archive (SRA) database under BioProject ID PRJNA721470.

## Supplementary Materials

The following are available online at https://www.mdpi.com/article/10.3390/v13091827/s1, Figure S1: Zika virus NGS coverage, Figure S2: Porcine circovirus 2 NGS coverage, File S1: PCV2 DNA, File S2: iVar protocol, Table S1: ZIKV and PCV2 PrimalSeq primers, Table S2: ZIKV and PCV2 in utero infection data, Table S3: ZIKV and PCV2-infected samples and NGS overview, Table S4: ZIKV and PCV2 SNVs, Table S5: NGS coverage gaps in the ZIKV genome, Table S6: In utero-emerging ZIKV and PCV2 iSNVs previously reported in field samples.
