# Supplementary material for: The Isolated in Utero Environment Is Conducive to the Emergence of RNA and DNA Virus Variants
Source: Viruses. 2021 Sep 14;13(9):1827. doi: 10.3390/v13091827 (PMC8473323; doi:10.3390/v13091827)
Supplement: Supplementary file 1 [file viruses-13-01827-s001.zip › File S1 - iVar protocol.pdf]

File S1 - iVar protocol

Single nucleotide variation analysis in Zika virus (ZIKV) and porcine circovirus 2 (PCV2) genomes

1 Summary

This manual describes a bioinformatics pipeline used to identify SNVs (single nucleotide variants) in Next-Generation Sequencing (NGS) data from ZIKV and PCV2 genomes. The pipeline can be adapted to analyse NGS data from other viruses.

In the first step, paired-end reads from illumina NGS data are preprocessed. After confirming the expected coverage and depth in the PhiX sequencing control, SNVs are detected with iVAR package.

2 Prerequisites

We use Linux for **data preprocessing**. UGENE for Windows/Linux can be used for the same analysis but has some issues preserving file names at batch processing of files.

Run all commands in the console/terminal/shell (**Show Applications button in the lowest left corner > Search for "Terminal" or "Konsole"**). Configure your Linux shell. Go to the **Terminal preferences > Unnamed profile > Scrolling >** Remove the checkmark from "Limit scrollback to ...".

For running each command, you need to enter the proper directory with the terminal session. For example, for trimming PhiX, you will need to use the directory **/home/ngs1/zika virus in utero heterogeneity IP versus IC/temporary**. To enter it with terminal, execute the following command:

```
cd "/home/ngs1/zika virus in utero heterogeneity IP versus IC/temporary"
```

After running each command:

- If several commands were copy-pasted, check that the last command was executed. If not, press Enter to launch the last command.
- Read all output in Terminal to check if there are no errors.
- Copy-paste the terminal session's output to a file (**log.txt**) and save it in the working folder for each step.

For each step, you need an empty working directory. Bring input files; after successful execution, delete input files, but preserve all newly created files (results) and reference

files (e.g., **GENOME**-, **BED**-files, etc.). This allows us to go back and to repeat any step under the same or different conditions, if necessary.

Create subfolders for each analysis step:

Step		Subfolder name for each analysis step	Comments
Section # in this manual	Name		
**na**	Raw data	**/0 raw data**	
**na**	Working directory for temporary files	**/temp**	
**4**	Quality control check-up	**/1 QC MiSeq**	
**5**	Processing of the PhiX control	**/2 QC PhiX**	
**5.1**	Trimming PhiX	**/2 QC PhiX/Trimmed**	
**5.2**	Aligning PhiX to reference	**/2 QC PhiX/BWA**	
**6.1**	Trimming ZIKV	**/3 Trimmed**	
**6.2**	Aligning ZIKV to reference	**/4 BWA**	
**6.3**	Coverage analysis	**/5 Coverage**	
**6.4**	Merge files representing one sample but pool #1 and pool #2	**/5.1 Merged**<br>**/5.2 Coverage**	This step can be optional, if pools were mixed prior to barcoding.
**6.5**	iVAR processing	**/6 iVAR**	
**6.6**	Coverage	**/7 Coverage**	
**6.7**	SNVs processing	**/8 SNV**	

3 Prepare the Linux environment

The step-by-step installation manual for iVAR is available online (https://andersen-lab.github.io/ivar/html/installpage.html).

First install all require dependencies:

```
sudo apt autoremove
sudo apt install autotools-dev gcc zlib1g-dev libbz2-dev \
liblzma-dev make libncurses5-dev autoconf g++ bwa r-base \
bedtools parallel default-jre mc krusader krename kate gawk
```

To install HTSlib libraries, download HTSlib 1.10 (1 Mb; http://www.htslib.org/download/), then finish the installation procedure.

```
tar -xvf ./htslib-1.10.tar.bz2
cd ./htslib-1.10/
```

```
./configure
make
sudo make install
```

If HTSlib is installed in a non-standard location (e.g.,**/usr/local/lib/**) and iVAR cannot find it, please add the following line to your BASH configuration file (**~/.bash_profile** or **~/.bashrc**) so that iVar can find HTSlib dynamic libraries during runtime. No restart is needed.

```
export LD_LIBRARY_PATH=$LD_LIBRARY_PATH:/usr/local/lib/
```

To install SAMlib libraries, download SAMtools 1.10 (4 Mb; *http://www.htslib.org/download/*) then finish the installation procedure.

```
tar -xvf ./samtools-1.10.tar.bz2
cd ./samtools-1.10/
./configure
make
sudo make install
```

Install iVAR (*https://github.com/andersen-lab/ivar*). Download iVAR 20200605 (1 Mb; *https://github.com/andersen-lab/ivar*) then finish the installation procedure.

```
unzip ./ivar-master.zip
cd ./ivar-master/
./autogen.sh
./configure
make
sudo make install
```

Test installation of iVAR 1.2

```
ivar version
```

Install Trimmomatic (v 0.39; binary; *http://www.usadellab.org/cms/?page=trimmomatic*). Download and unpack it to **/home/ngs1/soft/Trimmomatic-0.39/**. UGENE on Windows contains a build-in version of Trimmomatic and can replace a Linux version.

```
unzip ./Trimmomatic-0.39.zip
```

To install RStudio, download from the official website (*https://rstudio.com/products/rstudio/download/#download*) version for Ubuntu 18 (or your version) (*https://download1.rstudio.org/desktop/bionic/amd64/rstudio-1.2.5033-amd64.deb*). Then install the downloaded file (**Right mouse button > Open With Software Install > Install**)

4 Quality control

Use Illumina Seq Analysis Viewer 2.4.7.0 on Windows to see the general report of the sequencing (**Browse > Use MiSeq output whole folder as input for Illumina Seq Analysis Viewer**). The full manual for the software package is available at illumina's website (*https://support.illumina.com/content/dam/illumina-support/documents/documentation/software_documentation/sav/sequencing-analysis-viewer-user-guide-15020619-f.pdf*).

Create a report in Word containing charts and data. To enlarge the chart click the chevron in the upper right corner of each chart. To copy-paste charts, click it with the right mouse button and select **Copy To Clipboard**.

1. **Analysis tab**
    a. Data by Cycle
        i. **Error Rate** (percentage of wrong bases in the PhiX control reads).
        ii. **Called Int** (corrected intensity of base calls)
    b. **QScore Distribution** (Q20 corresponds to 1% error rate, Q30 corresponds to 0.1% error rate)
    c. Data by Lane
        i. **% Aligned** (Frequency of reads representing PhiX)
    d. **Qscore Heatmap** (Q-scores by cycle)
2. **Summary tab.** Press **Copy To Clipboard** to copy-paste data.
    a. **Total – Yield Total (G)** (Total number of Gbp saved)
    b. **Total – Aligned (%)** (Frequency of reads representing PhiX)
    c. **Error Rate (%)** (percentage of wrong bases in the PhiX control reads)
3. **Indexing tab.**
    a. **Total reads** (total number of reads saved)
    b. **% reads Identified (PF)** (percentage of reads with barcodes)
    c. **Min** and **Max** (percentage of the least and most frequent barcodes)
    d. Click on a table with individual indexes with left mouse button, then press **Control+A > Control+C** to copy-paste the table content.
    e. **Barchart** contains a graphical representation of the table. Copy-paste chart by clicking it with the right mouse button and selecting **Copy To Clipboard**.

Use the corresponding folder to save the results (screenshots).

5 Processing of the PhiX control

5.1 Trim adaptors and do quality trim with Trimmomatic

From the illumina machine output folder, copy-paste to a new working folder **2** PhiX **FASTQ.GZ** files containing non-barcoded paired reads that should include PhiX.

- **./0 raw/191125_M04229_0101_000000000-CRM9R/Data/Intensities/BaseCalls/Undetermined_S0_L001_R1_001.fastq.gz**
- **./0 raw/191125_M04229_0101_000000000-CRM9R/Data/Intensities/BaseCalls/Undetermined_S0_L001_R2_001.fastq.gz.**

Run Trimmomatic.

- This workflow is only valid for the paired-ended reads (**PE**). You cannot use it for single-ended (**SE**) reads.
- Workflow parameters: **ILLUMINACLIP:TruSeq3-PE-2.fa:2:30:10 LEADING:3 TRAILING:3 SLIDINGWINDOW:4:20 MINLEN:36**
- Illimina HiSeq is using Phred33 quality scores and needs **TruSeq3-PE-2.fa** file from the Trimmomatic package for removing adaptors. Input file for adaptors may vary depending on machine, etc. Check it each time.
- Phred >20 parameter used for quality trim corresponds to 99% accuracy in nucleotides.
- Adapt the directory pathway for the Trimmomatic package (highlighted) if necessary.

```
clear
date
mkdir trimmed
FOLDER=/home/ngs1/soft/Trimmomatic-0.39
TOTAL_FILES=`find -iname '*.fastq.gz' | wc -l`
ARR=($(ls *.fastq.gz))
for ((i=0; i<$TOTAL_FILES; i+=2))
{
SAMPLE_NAME=`echo ${ARR[$i]} | awk -F "_" '{print $1}'`
printf "\n"
echo "[trimming] $SAMPLE_NAME"
echo "java -jar $FOLDER/trimmomatic-0.39.jar PE ${ARR[$i]} ${ARR[$i+1]} -baseout ./trimmed/$SAMPLE_NAME.fastq.gz
ILLUMINACLIP:$FOLDER/adapters/TruSeq3-PE-2.fa:2:30:10 LEADING:3 TRAILING:3 SLIDINGWINDOW:4:20 MINLEN:36"
java -jar $FOLDER/trimmomatic-0.39.jar PE ${ARR[$i]} ${ARR[$i+1]} -baseout ./trimmed/$SAMPLE_NAME.fastq.gz
ILLUMINACLIP:$FOLDER/adapters/TruSeq3-PE-2.fa:2:30:10 LEADING:3 TRAILING:3 SLIDINGWINDOW:4:20 MINLEN:36
}
cd trimmed
mkdir paired; mv *P.fastq.gz paired
mkdir unpaired; mv *U.fastq.gz unpaired
cd ..
date
```

After trimming, check the report of Trimmomatic carefully for errors and save it as a log file (**log.txt**). The output is **4** files sorted in two directories:

- 2 files with paired reads (forward and reverse): **<SAMPLE>_1P.fastq.gz** and **<SAMPLE>_2P.fastq.gz** stored in **./trimmed/paired** directory.
- 2 files with unpaired reads (forward and reverse): **<SAMPLE>_1U.fastq.gz** and **<SAMPLE>_2U.fastq.gz** stored in **./trimmed/unpaired** directory.

Collect and keep generated output files from the newly created folder named '**trimmed**'. For the following steps, you will need only **2** files with paired reads.

5.2 Align reads to the reference

Make a copy of output files from Trimmomatic corresponding to paired reads (2 files per sample). For the PhiX control sample name is **Undetermined**.

Provide a file representing the reference for the alignment.

- **This is an important step!** Get the correct reference (**FASTA file**) for PhiX from the illumina website (*https://support.illumina.com/sequencing/sequencing_software/igenome.html*). Genbank has the PhiX sequence (**NC_001422.1**) that is 5 nt different from the illumina's one.

Place the reference **FASTA** file in the same folder as **FASTQ.GZ** files from Trimmomatic. Then make index files for the reference genome (highlighted).

```
bwa index illumina-phix.fa
```

To process **paired** reads, use the following commands:

- When using another reference genome, change index name (highlighted in yellow).

```
date
INDEX=illumina-phix.fa
TOTAL_FILES=`find -name '*.fastq.gz' | wc -l`
ARR=($(ls *.fastq.gz))
for ((i=0; i<$TOTAL_FILES; i+=2))
{
printf "\n"
SAMPLE_NAME=`echo ${ARR[$i]} | awk -F "_" '{print $1}'`
echo "[mapping running for $[i/2+1]/$[TOTAL_FILES/2]] $SAMPLE_NAME"
echo "bwa mem -t 32 $INDEX ${ARR[$i]} ${ARR[$i+1]} | samtools view -b -F 4 -F 2048 | samtools sort -o $SAMPLE_NAME.bam"
bwa mem -t 32 $INDEX ${ARR[$i]} ${ARR[$i+1]} | samtools view -b -F 4 -F 2048 | samtools sort -o $SAMPLE_NAME.bam
}
date
```

The resulting output is a **sorted and merged BAM file** named as **<SAMPLE>. BAM**. Unmapped reads are skipped ("F" parameter). After successfully finalizing the run, delete the copy-pasted input **FASTQ.GZ** files to save space, but keep the generated output **BAM file** and logging information from the shell.

5.3 Coverage

To visually inspect the coverage, open the resulting BAM-file with UGENE.

PhiX genome should be covered fully (100% genome coverage) with a high coverage depth (>10'000x).

6 SNV ANALYSIS IN ZIKV GENOME

6.1 Trim adaptors and do quality trim with Trimmomatic

Do it exactly as for PhiX, but use **FASTQ.GZ** files corresponding to the experimental samples from the illumina machine output folder. Similarly to PhiX processing, after successful trimming, you will need only **2** files with paired reads for the following steps.

6.2 Align reads to the reference

This step is done exactly as for PhiX analysis. Make a copy of output files from Trimmomatic corresponding to paired reads (2 files per sample). The only difference is the reference genome provided. Similarly to PhiX processing, don't mix paired and unpaired files after Trimmomatic. Proceed only with paired ones. Don't forget to save the log file from Terminal.

- **This is an important step!** Don't forget to provide the **CORRECT** reference genome. Get the correct reference (**FASTA file**) (e.g., for the PR strain of ZIKV from the Genbank (**KU501215.1**)).

The resulting output is a **sorted and merged BAM file** named as **<SAMPLE>. BAM**.

6.3 Coverage

To visually inspect the coverage of any sample, open the resulting **BAM-file** with UGENE.

For batch processing of all samples at once and detailed analysis:

1. Create a **GENOME** file from the reference **FASTA** file (e.g., **zika-pr.fasta**).

```
INDEX=zika-pr.fasta
samtools faidx $INDEX
awk {'printf $1 "\t" $2'} $INDEX.fai > $INDEX.genome
```

2. Copy the final (merged and sorted) **BAM-files** to a new directory. Place it in the same folder where the correct **GENOME** file (highlighted in yellow). The following commands will produce **TXT** files with the coverage. Besides this **BED** files are created. You may delete them as soon as they will not be required further.

```
find ./ -iname '*.bam' | parallel -j 4 --progress 'bedtools bamtobed -i {} > {.}.bed | echo {/}'
find ./ -iname '*.bed' | parallel -j 4 --progress 'bedtools genomecov -d -i {} -g zika-pr.fasta.genome > {.}.txt'
```

3. Analyse resulting files with R script.
    a. Create the R script (**coverage.Rmd**) containing following code.

```
---
title: "Coverage parsing"
author: "Ivan Trus"
date: "2020-10-19"
```

```
output:
  html_document: default
  pdf_document: default
---
Execution start time: *`r Sys.time()`*
```{r setup, include=FALSE}
knitr::opts_chunk$set(
  echo = FALSE,
  message = TRUE,
  warning = TRUE
)
library(vroom)
library(reshape)
```

Initialization of the script and data loading
```{r init, message=FALSE}
Filelist <- list.files(pattern = ".*.txt")
CountGenomes <- length(Filelist)
Threshold <- 400

# Defining functions
ListGaps <- function(Gaps) {
  Start <- append(Gaps[1], Gaps[which(diff(Gaps) > 1) + 1])
  Start
  End <- append(Gaps[which(diff(Gaps) > 1)], Gaps[length(Gaps)])
  End
  GapsSummary <- data.frame(Start, End)
  colnames(GapsSummary) <- c("Start", "End")
  return(GapsSummary)
}

Coverage <- vroom(Filelist, id = Filelist, col_names = "")
Coverage <- Coverage[c(1, 3, 4)]
colnames(Coverage)[1] <- "X1"
Coverage <- t(cast(Coverage, X1 ~ X2, value.var = "X3"))
colnames(Coverage) <- gsub(".txt", "", Filelist)
GenomeLength <- length(Coverage[, 1])

```

* Current working directory: **`r getwd()`**
* List of files to process: **`r Filelist`**
* Total number of files: **`r CountGenomes`**
* Threshold for coverage counting: **`r Threshold`** x
* Genome length: **`r GenomeLength`** nt

Diagnostic data, chart and list of gaps for each sample
```{r main}
Results <- matrix(
  nrow = CountGenomes, ncol = 3,
  dimnames = list(1:CountGenomes, c(
    "Sample", "Read depth, x", "Coverage, %"))
)
Results <- as.data.frame(Results)
Results[, 1] <- colnames(Coverage)
AllGaps <- NA

summary(Coverage)

for (i in 1:CountGenomes) {
  Results[i, 2] <- round(mean(Coverage[, i]), digits = 4)
  Results[i, 3] <- round(100 - sum(Coverage[, i] < Threshold) / GenomeLength * 100, digits = 4)

  # We need to replace 0 with 1 to make it possible to be plotted fully on log scale
  GapsForLogChart <- Coverage[, i]
  GapsForLogChart[GapsForLogChart == 0] <- 1

  if(max(GapsForLogChart) < Threshold) YaxisLimit <- Threshold else YaxisLimit <- max(GapsForLogChart)
  plot(log10(GapsForLogChart),
```
```

```
    type = "l", xlab = "Nucleotide position in the ZIKV genome",
    ylab = "Read depth (log10)", ylim = c(0, log10(YaxisLimit) * 1.05))
  text(x = 0, y = 1.05 * log10(YaxisLimit), colnames(Coverage)[i], pos = 4, font = 2, cex = 1.2)
  abline(h = log10(Threshold), col = "red", lwd = 3)
  GapsPositions <- as.numeric(names(subset(Coverage[, i], Coverage[, i] < Threshold)))
  AllGaps <- sort(c(AllGaps, setdiff(GapsPositions, AllGaps)))
  print(paste("The list of gaps in", colnames(Coverage)[i], "with the depth less than", Threshold, "x"))
  print(ListGaps(GapsPositions))
}
```

Summary on read depth and coverage
```{r final}
Results
print(paste("Combined gaps with coverage depth less than", Threshold, "x."))
print(ListGaps(AllGaps))
print(paste("Intersection of coverage in all provided files:", round(100 - length(AllGaps) / GenomeLength * 100, digits = 4),
"%"))
```

Execution end time: *`r Sys.time()`*
```

b. X and Y graph axis titles are highlighted and can be adapted as needed.
c. Place the created R script (**coverage.Rmd**) and **TXT** files with coverages to a separate (new) directory. Make sure that only the relevant **TXT** files were placed in this folder.
d. Open and run the R script with RStudio.
    i. Use **Code > Run Region > Run All (Control+Alt+R)** if you want to see results in RStudio.
    ii. Use **File > Knit Document** if you need a report (**HTML** file) generated. This file can be opened with any web browser. To create a report in **PDF** file format, you need first to install the LaTeX distribution.
    iii. If there is an Error: "*Line 22 Error in loadnamesspace(I, c(lib.loc, .libPaths()), versioncheck = vI[[i]]) : namespace 'rlang' 0.4.6 is already loaded, but >= 0.4.7 is required calls: &lt;Anonymous&gt; … getNamespace -> namespaceImport -> loadNamespace*", update '**rlang**' package in R studio.

## 6.4 Merge files representing one sample but pool #1 and pool #2

Do this step when DNA representing the whole ZIKV or PCV2 genome (amplified with two different primer pools; see materials and methods for PrimalSeq NGS) were not mixed during NGS library preparation. If pools were mixed, each biological sample is represented by one BAM file only, and merging is not required. **Pools are not the same as technical replicates**.

1. Rename all files manually. E.g. for Pool #1 and Pool #2: files **4A.bam** and **5A.bam** should be renamed to **4A.bam** and **4A_5A.bam**.
2. The following commands process pair of files at once. Thus, temporarily remove singlets (negative control samples represented by pool #1 or pool #2 only) to a separate folder.

```
mkdir 1-merged
mkdir 2-sorted
```
```

```
TOTAL_FILES=`find -iname '*.bam' | wc -l`
ARR=($(ls *.bam))
for ((i=0; i<$TOTAL_FILES; i+=2))
{
SAMPLE_NAME=`echo ${ARR[$i]} | awk -F "_" '{print $1}'`
printf "\n"
echo "[merging] $SAMPLE_NAME"
echo "samtools merge ./1-merged/$SAMPLE_NAME.bam ${ARR[$i]} ${ARR[$i+1]}"
samtools merge ./1-merged/$SAMPLE_NAME.bam ${ARR[$i]} ${ARR[$i+1]}
}
cd 1-merged
TOTAL_FILES=`find -iname '*.bam' | wc -l`
ARR=($(ls *.bam))
for ((i=0; i<$TOTAL_FILES; i+=1))
{
SAMPLE_NAME=`echo ${ARR[$i]} | awk -F "_" '{print $1}'`
printf "\n"
echo "[sorting] $SAMPLE_NAME "
echo "samtools view -b ${ARR[$i]} | samtools sort -o ./../2-sorted/$SAMPLE_NAME"
samtools view -b ${ARR[$i]} | samtools sort -o ./../2-sorted/$SAMPLE_NAME
}
cd ..
```

3. Bring singlets back to merged ones.
4. Calculate coverage after merging exactly as in step 6.3.

6.5 iVAR processing

Place in one directory

- final (sorted and merged) BAM files
    - The following commands process pair of files at once. Thus, it should be two technical replicates per sample (two BAM files) only. No more, no less.
    - Rename all these replicates to have a similar beginning of the filename ending with "_" symbol. E.g for **10A** sample keep "**10A_**" as first symbols: **10A_r1.BAM** and **10A_r2.BAM**. **10A_.BAM** and **10A_1.BAM** are also fine. Any file like **10A._BAM** or **10A.BAM** will be wrong.
- FASTA file (**zika_primers.fa**) with ZIKV primers which were used for targeted amplification of pool #1 and pool #2
- FASTA file with the ZIKV reference genome (**zikv-pr.fasta**). The same one as in step 6.2.
- TSV file with information on ZIKV primer pairs (**zika_primer_pair_information.tsv**). This file should be created manually in the text editor.
    - Example primer pair information file from iVAR manual (*https://github.com/andersen-lab/ivar/blob/master/docs/MANUAL.md*)

```
400_1_out_L	400_1_out_R
400_2_out_L	400_2_out_R
400_3_out_L	400_3_out_R
...
```

- GFF file with information on ORFs (**zikv-pr.GFF3**).
    - To make GFF file, go to Genbank and find your reference genome (**KU501215.1** for ZIKV).

- Export GFF file: **Send to: > Complete Record > Choose Destination: File > Format: GFF3 > Create file**
- Don not pay attention to the message “GFF file is not in GFF3 file format!” in the final iVar output. According to iVar author (https://github.com/andersen-lab/ivar/issues/23):
    - *The GFF3 file from NCBI has 4 lines starting with #! and those lines are showing the error. It's a simple fix and this should be done for next release. Irrespective of that though, ivar should proceed with translation as expected.*

Note: Be very cautious because GFF works with ZIKV but did not work with PCV2. In PCV2 genome ORF2 transcribed in reverse direction and GFF gives misleading positions for AA. Be careful working with viruses with genomes different from flaviviruses; for correct identification of AA positions use manual method.

Run script that executes the following steps:

1. Creating the main **BAM file with primers**. Creating the main **BED file with primers**.
2. Counting and processing **BAM files with replicates** of sample reads one-by-one. **BAM file with primers** is not counted and is not processed in this pipeline.
    e. Making index.
    f. Trimming (soft clipping) primers from BAM.
    g. Sorting new BAM. Indexing BAM. Merging replicates to a **merged BAM file**.
    h. Processing **merged BAM file** representing both replicates.
        i. Making **consensus FASTA file** from **merged BAM**. Indexing it. Aligning primers to it and creating **individual sorted BAM files with primers** for each sample.
        ii. Creating the **TSV file with masked primers**. Creating **individual BED file with primers**.
    i. Removing reads with mismatches in primer sequences from **BAM files representing replicates**. Sorting of resulting BAM files.
    j. Generating the final **TSV file with the list of mutations** in each replicate. Only mutations above the threshold (3%; highlighted with red in the script) are recorded.
    k. Filtering similar **SNVs** across replicates. Generating the final **TSV file with the list of mutations** in a sample (in each of both replicates).

- Input files are highlighted with yellow. If you have different ones then edit the highlighted fragments accordingly.

```
clear
date
REFERENCE=zikv-pr.fasta
PRIMERS=zika_primers.fa
PRIMER_PAIRS=zika_primer_pair_information.tsv
GFF_FILE=zikv-pr.gff3
# We are counting original BAM files here because one extra BAM file will be created soon for primers.
TOTAL_FILES=`find -iname '*.bam' | wc -l`
ARR=($(ls *.bam))
bwa index $REFERENCE
bwa mem -k 5 -T 16 $REFERENCE $PRIMERS | samtools view -b -F 4 > primers.bam
bedtools bamtobed -i primers.bam > primers.bed
printf "\n\n"
```

```
        echo "Part 1: processing individual files with indexing, trimming, sorting, and indexing"
        printf "\n"
        for ((i=0; i<$TOTAL_FILES; i+=1)) {
          FILE_NAME=`echo ${ARR[$i]} | awk -F "." '{print $1}'`
          printf "\n"
          echo "[processing] $FILE_NAME"
          echo "[indexing] samtools index $FILE_NAME.bam"
          samtools index $FILE_NAME.bam
          echo "[trimming] ivar trim -b primers.bed -i ${ARR[$i]} -p $FILE_NAME.trimmed"
          ivar trim -b primers.bed -i ${ARR[$i]} -p $FILE_NAME.trimmed
          echo "[sorting] samtools sort $FILE_NAME.trimmed.bam -o $FILE_NAME.trimmed.sorted.bam"
          samtools sort $FILE_NAME.trimmed.bam -o $FILE_NAME.trimmed.sorted.bam
          echo "[indexing] samtools index $FILE_NAME.trimmed.sorted.bam"
          samtools index $FILE_NAME.trimmed.sorted.bam
        }
        printf "\n\n"
        echo "Part 2: processing two replicates with merging, making and indexing consensus, primers processing (aligning, framing, and finding mismatches)"
        printf "\n"
        for ((i=1; i<$TOTAL_FILES; i+=2)) {
          SAMPLE_NAME=`echo ${ARR[$i]} | awk -F "_" '{print $1}'`
          FILE_NAME1=`echo ${ARR[$i-1]} | awk -F "." '{print $1}'`
          FILE_NAME2=`echo ${ARR[$i]} | awk -F "." '{print $1}'`
          printf "\n"
          echo "[processing] $SAMPLE_NAME"
          echo "[merging replicates] samtools merge $SAMPLE_NAME.merged.bam $FILE_NAME1.trimmed.sorted.bam $FILE_NAME2.trimmed.sorted.bam"
          samtools merge $SAMPLE_NAME.merged.bam $FILE_NAME1.trimmed.sorted.bam $FILE_NAME2.trimmed.sorted.bam
          echo "[making consensus] samtools mpileup -A -d 1000000 -Q 0 $SAMPLE_NAME.merged.bam | ivar consensus -p $SAMPLE_NAME.consensus"
          samtools mpileup -A -d 1000000 -Q 0 $SAMPLE_NAME.merged.bam | ivar consensus -p $SAMPLE_NAME.consensus
          echo "[primers processing: indexing consensus] bwa index $SAMPLE_NAME.consensus.fa"
          bwa index $SAMPLE_NAME.consensus.fa
          echo "[primers processing: conversion to sorted bam] bwa mem -k 5 -T 16 $SAMPLE_NAME.consensus.fa $PRIMERS | samtools view -bS -F 4 | samtools sort -o $SAMPLE_NAME.primers_consensus.bam"
          bwa mem -k 5 -T 16 $SAMPLE_NAME.consensus.fa $PRIMERS | samtools view -bS -F 4 | samtools sort -o $SAMPLE_NAME.primers_consensus.bam
          echo "[primers processing: creating BED] bedtools bamtobed -i $SAMPLE_NAME.primers_consensus.bam > $SAMPLE_NAME.primers_consensus.bed"
          bedtools bamtobed -i $SAMPLE_NAME.primers_consensus.bam > $SAMPLE_NAME.primers_consensus.bed
          echo "[primers processing: creating TSV file] samtools mpileup -A -d 1000000 --reference $SAMPLE_NAME.consensus.fa -Q 0 $SAMPLE_NAME.primers_consensus.bam | ivar variants -p $SAMPLE_NAME.primers_consensus -t 0.03"
          samtools mpileup -A -d 1000000 --reference $SAMPLE_NAME.consensus.fa -Q 0 $SAMPLE_NAME.primers_consensus.bam | ivar variants -p $SAMPLE_NAME.primers_consensus -t 0.03
          echo "[primers processing: masking] ivar getmasked -i $SAMPLE_NAME.primers_consensus.tsv -b $SAMPLE_NAME.primers_consensus.bed -f $PRIMER_PAIRS -p $SAMPLE_NAME.mismatches"
          ivar getmasked -i $SAMPLE_NAME.primers_consensus.tsv -b $SAMPLE_NAME.primers_consensus.bed -f $PRIMER_PAIRS -p $SAMPLE_NAME.mismatches
        }
        printf "\n\n"
        echo "Part 3: processing individual files with removing reads with mismatches, sorting BAM-file, and calling SNVs"
        printf "\n"
        for ((i=0; i<$TOTAL_FILES; i+=1)) {
          SAMPLE_NAME=`echo ${ARR[$i]} | awk -F "_" '{print $1}'`
          FILE_NAME=`echo ${ARR[$i]} | awk -F "." '{print $1}'`
          printf "\n"
          echo "[processing] $FILE_NAME"
          echo "[removing reads] ivar removereads -i $FILE_NAME.trimmed.sorted.bam -p $FILE_NAME.trimmed.sorted.masked.bam -t $SAMPLE_NAME.mismatches.txt -b primers.bed"
          ivar removereads -i $FILE_NAME.trimmed.sorted.bam -p $FILE_NAME.trimmed.sorted.masked.bam -t $SAMPLE_NAME.mismatches.txt -b primers.bed
          echo "[sorting resulting BAM] samtools sort $FILE_NAME.trimmed.sorted.masked.bam -o $FILE_NAME.trimmed.sorted.masked.sorted.bam"
          samtools sort $FILE_NAME.trimmed.sorted.masked.bam -o $FILE_NAME.trimmed.sorted.masked.sorted.bam
          echo "[generating final TSV file] samtools mpileup -A -d 1000000 --reference $REFERENCE –B -Q 0 $FILE_NAME.trimmed.sorted.masked.sorted.bam | ivar variants -p $FILE_NAME.SNV -t 0.03 -r $REFERENCE -g GFF_FILE"
          samtools mpileup -A -d 1000000 --reference $REFERENCE –B -Q 0 $FILE_NAME.trimmed.sorted.masked.sorted.bam | ivar variants -p $FILE_NAME.SNV -t 0.03 -r $REFERENCE -g GFF_FILE
        }
        printf "\n\n"
```

```
        echo "Part 4: Filtering the same SNVs from replicates"
        printf "\n"
        for ((i=1; i<$TOTAL_FILES; i+=2)) {
           SAMPLE_NAME=`echo ${ARR[$i]} | awk -F "_" '{print $1}'`
           FILE_NAME1=`echo ${ARR[$i-1]} | awk -F "." '{print $1}'`
           FILE_NAME2=`echo ${ARR[$i]} | awk -F "." '{print $1}'`
           printf "\n"
           echo "[processing] $SAMPLE_NAME"
           echo "[filtering SNVs across replicates] ivar filtervariants -p $SAMPLE_NAME.SNV_final $FILE_NAME1.SNV.tsv
$FILE_NAME2.SNV.tsv"
           ivar filtervariants -p $SAMPLE_NAME.SNV_final $FILE_NAME1.SNV.tsv $FILE_NAME2.SNV.tsv
        }
        echo "[Sorting files to directories and renaming]"
        mkdir 0.input && mv $PRIMERS $REFERENCE $PRIMER_PAIRS $GFF_FILE $_
        mkdir 1.index_files && mv *.pac $_ && mv *.sa $_ && mv *.ann $_ && mv *.amb $_ && mv *.fai $_ && mv *.bai $_ && mv *.bwt
$_
        mkdir 1.Primers_info && mv primers.bam primers.bed $_
        mkdir 2a.Consensus && mv *.consensus.fa $_
        mkdir 2c.BED && mv *.bed $_
        mkdir 2d.Consensus-TSV && mv *.primers_consensus.tsv $_
        mkdir 3.Mismatches  && mv *.mismatches.txt $_
        mkdir 4.Quality && mv *.qual.txt $_
        mkdir 7.SNV-final && mv *.SNV_final.tsv $_
        mkdir 6.SNV-singlets && mv *.SNV.tsv $_
        mkdir 5.BAM-final && mv *.masked.sorted.bam $_
        rm *.masked.bam
        mkdir 2b.BAM-primers && mv *.primers_consensus.bam $_
        rm *.merged.bam
        rm *.sorted.bam
        rm *.trimmed.bam
        mkdir 0.BAM-original && mv *.bam $_
        cd ./7.SNV-final
        for FILE in *; do mv "$FILE" "${FILE/SNV_final./}"; done
        cd ../5.BAM-final
        for FILE in *; do mv "$FILE" "${FILE/trimmed.sorted.masked.sorted./}"; done
        cd ..
        date
        echo "[FINISHED]"
```

6.6 Analysis of low-frequency SNVs present in the virus inoculum

To confirm that *de novo* mutations emerged *in vivo* did not initially present in the virus inoculum, re-run the code provided in chapter 6.6 for virus inoculum samples. Change mutation detection threshold from 3% (0.03; highlighted with red color in the script) to 0.01% (0.0001).

Check the output file with the threshold of 0.1% for the mutations detected in your samples with the threshold of 3%. Use reference values for your equipment to assess importance of the detected mutations in the positive control stock/inoculum.

For illumina Miseq, company states for 0.1% (doi: 10.1111/j.1755-0998.2011.03024.x), while research publications report 0.24% (doi: 10.1038/s41598-018-29325-6) and 0.30-0.46% (doi: 10.1186/gb-2013-14-5-r51) as an error rate.

6.7 Calculate coverage for the final masked and sorted BAM files

Calculate coverage in final BAM files generated by iVAR exactly as in step 6.3.

6.8 SNVs processing

Import **final TSV files** to Microsoft Excel for Microsoft Windows (this will not work in Libre Office).

1. Put your **TSV** files in a dedicated folder.
2. Open Excel. Create new empty document.
3. **Data > New Query > From File > From Folder > <Provide the pathway for your folder with TSV files> OK > Combine > Combine&Edit > Change "Data Type Detection" to "Do not detect data types" and "Delimiter" to "Tab"> OK > File > Close&Load**
    a. At this step, mutations with any coverage are imported.
4. For each technical replicate delete **manually** lines representing insertions/deletions (i.e., mutations labeled with plus or minus sign. E.g. column E contains "**+A**").
5. For each technical replicate delete manually lines representing mutations with coverage <400 (Columns R and AB: **TOTAL_DP**).
6. Add the "**Group**" column and fill it with information on groups.

Optional. Use Microsoft Excel to find the masked regions (coverage <400) in the positive control sample and exclude mutations in all samples falling in these regions. Information about masked regions is available in the report of the R script generated in step 6.6.
