# Supplementary material for: The Isolated in Utero Environment Is Conducive to the Emergence of RNA and DNA Virus Variants
Source: Viruses. 2021 Sep 14;13(9):1827. doi: 10.3390/v13091827 (PMC8473323; doi:10.3390/v13091827)
Supplement: Supplementary file 1 [file viruses-13-01827-s001.zip › File S2 - PCV2 DNA.pdf]

File S2 - PCV2 DNA

Start with : CCGC,
PCV2 Sequence:
GGGCTGGCTGAACTTTTGAAAGTGAGCGGGAAAATGCAGAAGCGTGATTGGAAGACTAATGTACACGTCATTGTGGGGCCAC
CTGGGTGTGGTAAAAGCAAATGGGCTGCTAATTTTGCAGACCCGGAAACCACATACTGGAAACCACCTAGAAACAAGTGGTG
GGATGGTTACCATGGTGAAGAAGTGGTTGTTATTGATGACTTTTATGGCTGGCTGCCCTGGGATGATCTACTGAGACTGTGT
GATCGATATCCATTGACTGTAGAGACTAAAGGTGGAACTGTACCTTTTTTGGCCCGCAGTATTCTGATTACCAGCAATCAGA
CCCCGTTGGAATGGTACTCCTCAACTGCTGTCCCAGCTGTAGAAGCTCTTTATCGGAGGATTACTTCCTTGGTATTTTGGAA
GAATGCTACAGAACAATCCACGGAGGAAGGGGGCCAGTTCGTCACCCTTTCCCCCCCATGCCCTGAATTTCCATATGAAATA
AATTACTGAGTCTTTTTTATCACTTCGTAATGGTTTTTATTATTCATTAAGGGTTAAGTGGGGGGTCTTTAAGATTAAATTC
TCTGAATTGTACATACATGGTTACACGGATATTGTATTCCTGGTCGTATATACTGTTTTCGAACGCAGTGCCGAGGCCTACG
TGGTCTACATTTCCAGCAGTTTGTAGTCTCAGCCACAGCTGGTTTCTTTTGTTGTTTGGTTGGAAGTAATCAATAGTGGAAT
CTAGGACAGGTTTGGGGGTAAAGTAGCGGGAGTGGTAGGAGAAGGGCTGGGTTATGGTATGGCGGGAGGAGTAGTTTACATA
GGGGTCATAGGTGAGGGCTGTGGCCTTTGTTACAAAGTTATCATCTAGAATAACAGCACTGGAGCCCACTCCCCTGTCACCC
TGGGTGATCGGGGAGCAGGGCCAGAATTCAACCTTAACCTTTCTTATTCTGTAGTATTCAAAGGGCACAGAGCGGGGGTTTG
AGCCCCCTCCTGGGGGAAGAAAGTCATTAATATTGAATCTCATCATGTCCACCGCCCAGGAGGGCGTTCTGACTGTGGTTCG
CTTGATAGTATATCCGAAGGTGCGGGATAGGCGGGTGTTGAAGATGCCATTTTTCCTTCTCCAGCGGTAACGGTGGCGGGGG
TGGACGAGCCAGGGGCGGCGGCGGAGGATCTGGCCAAGATGGCTGCGGGGGCGGTGTCTTCTTTTCCGGTAACGCCTCCTTG
GATACGTCATATCTGAAAACGAAAGAAGTGCGCTGTAAGTATTACCAGCGCACTTCGGCAGCGGCAGCACCTCGGCAGCACC
TCAGCAGCAACATGCCCAGCAAGAAGAATGGAAGAAGCGGACCCCAACCCCATAAAAGGTGGGTGTTCACTCTGAATAATCC
TTCCGAAGACGAGCGCAAGAAAATACGGGATCTTCCAATATCCCTATTTGATTATTTTATTGTTGGCGAGGAGGGTAATGAG
GAAGGACGAACACCTCACCTCCAGGGGTTCGCTAATTTTGTGAAGAAGCAGACTTTTAATAAAGTGAAGTGGTATTTGGGTG
CCCGCTGCCACATCGAGAAAGCCAAAGGAACAGATCAGCAGAATAAAGAATACTGCAGTAAAGAAGGCAACTTACTGATTGA
GTGTGGAGCTCCTAGATCTCAGGGACAACGGAGTGACCTGTCTACTGCTGTGAGTACCTTGTTGGAGAGCGGGAGTCTGGTG
ACCGTTGCAGAGCAGTACCCTGTAACGTTTGTCAGAAATTTCCGC
End with : GG.
