# Supplementary figures and images for: The Isolated in Utero Environment Is Conducive to the Emergence of RNA and DNA Virus Variants
Source: Viruses. 2021 Sep 14;13(9):1827. doi: 10.3390/v13091827 (PMC8473323; doi:10.3390/v13091827)

Figure. S1 Zika virus NGS coverage

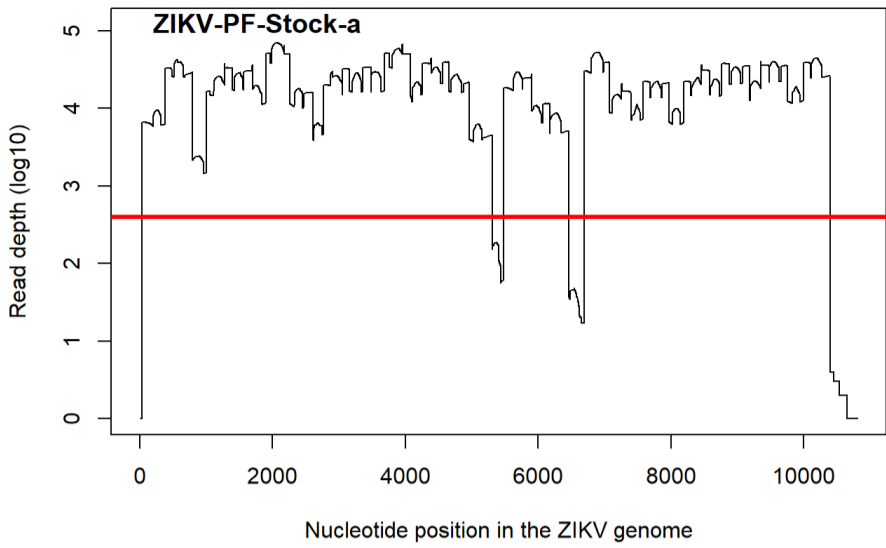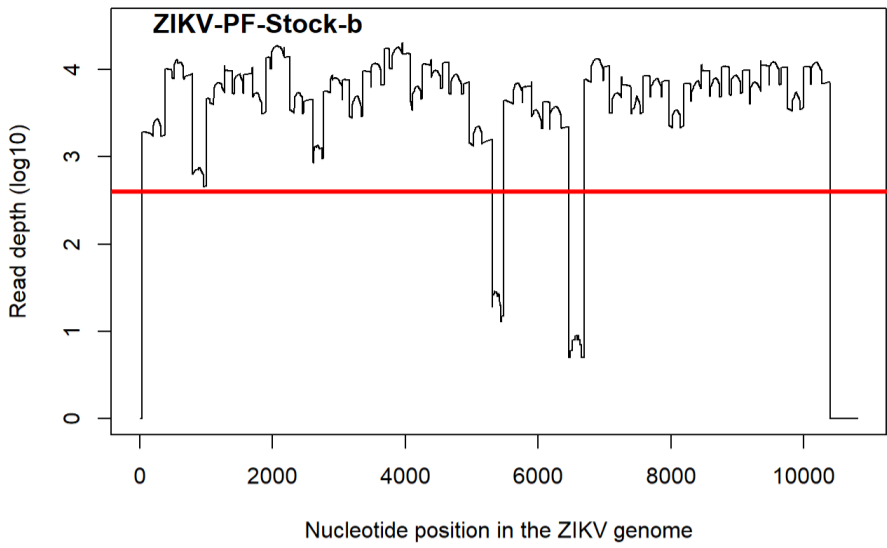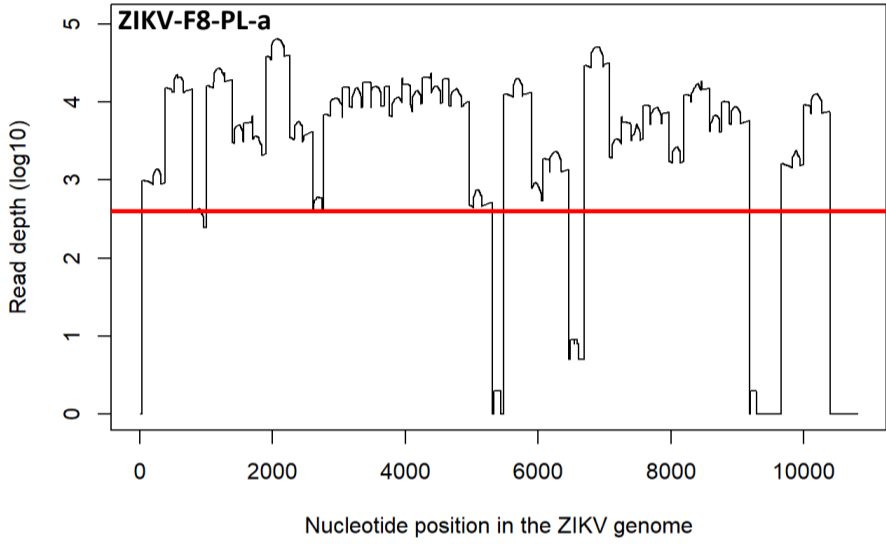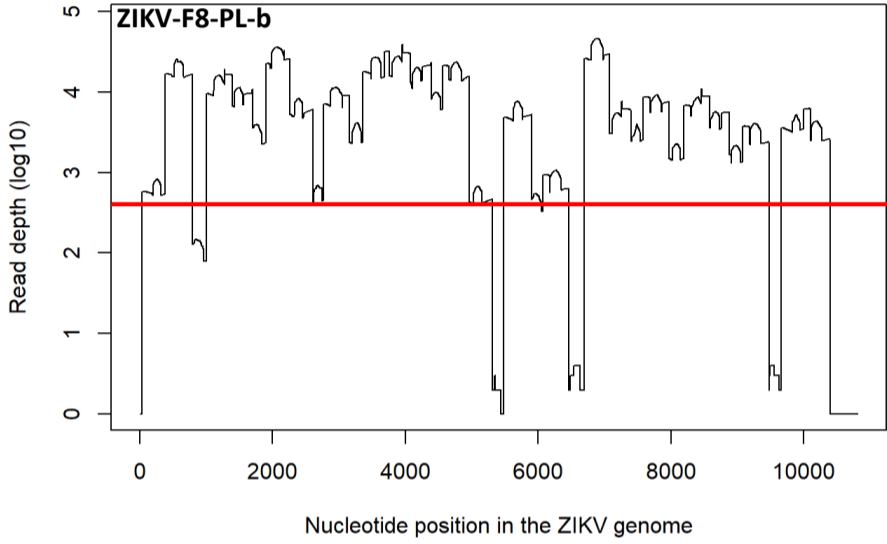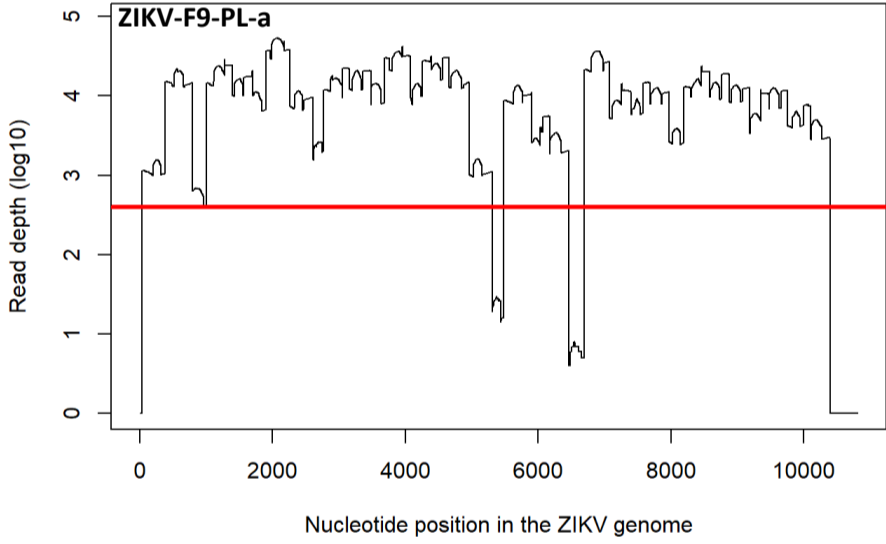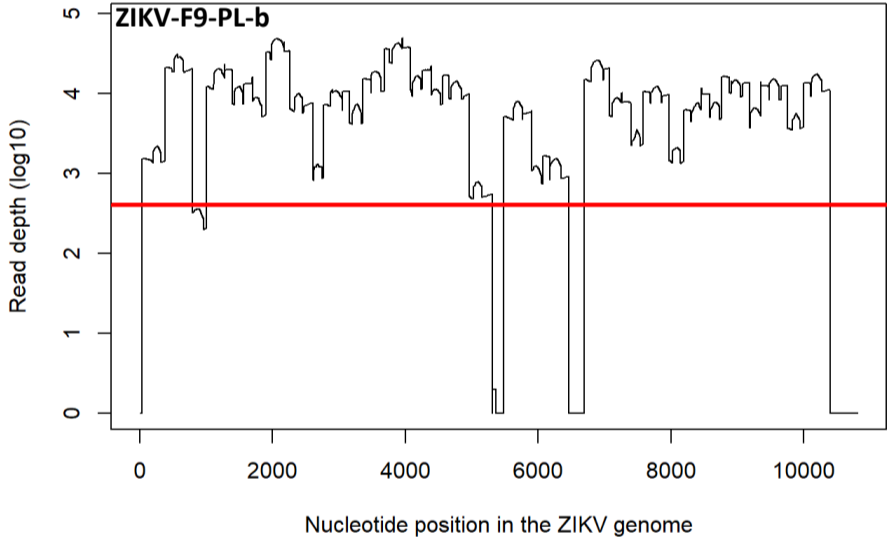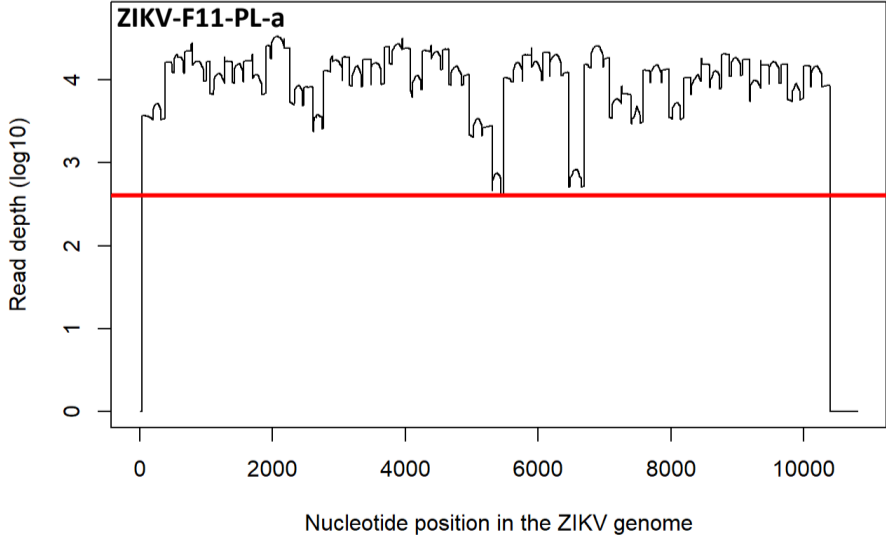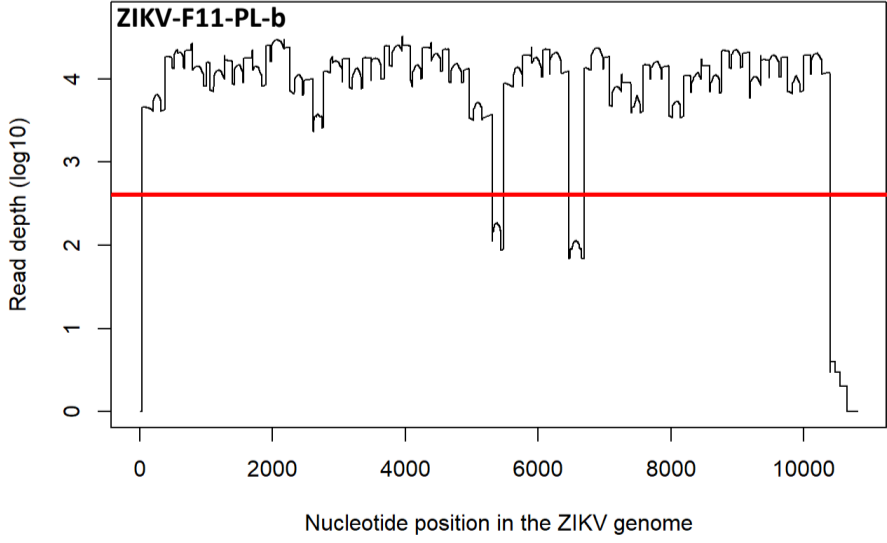

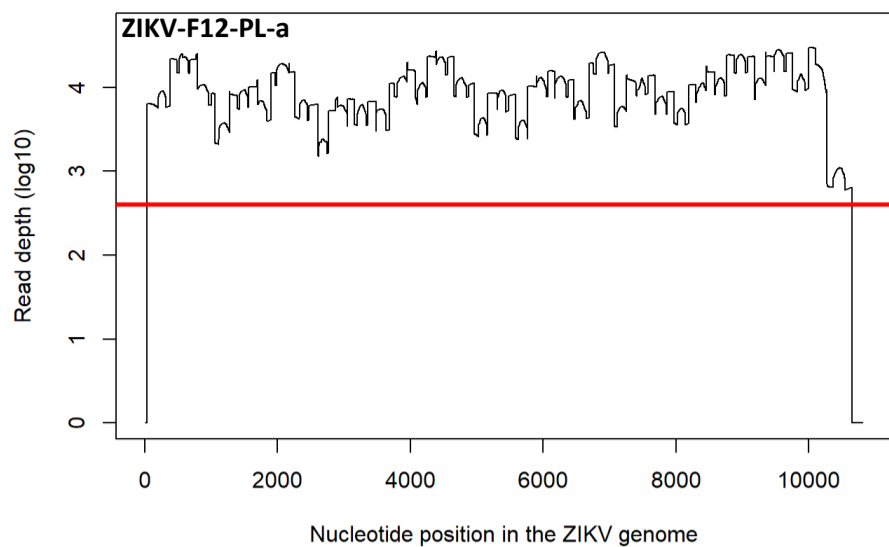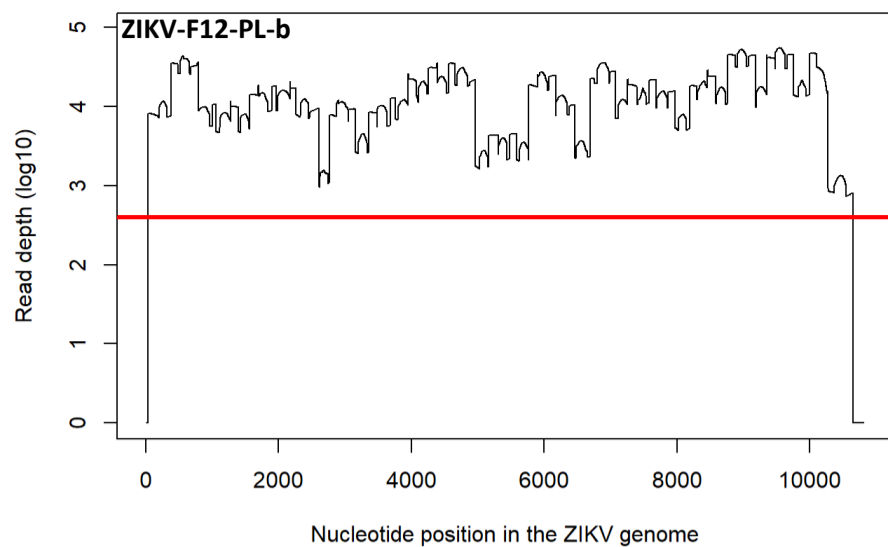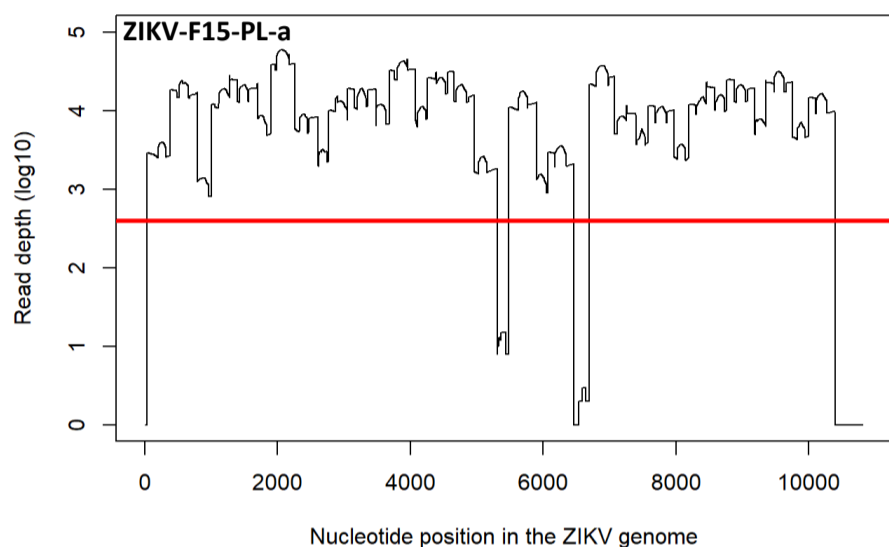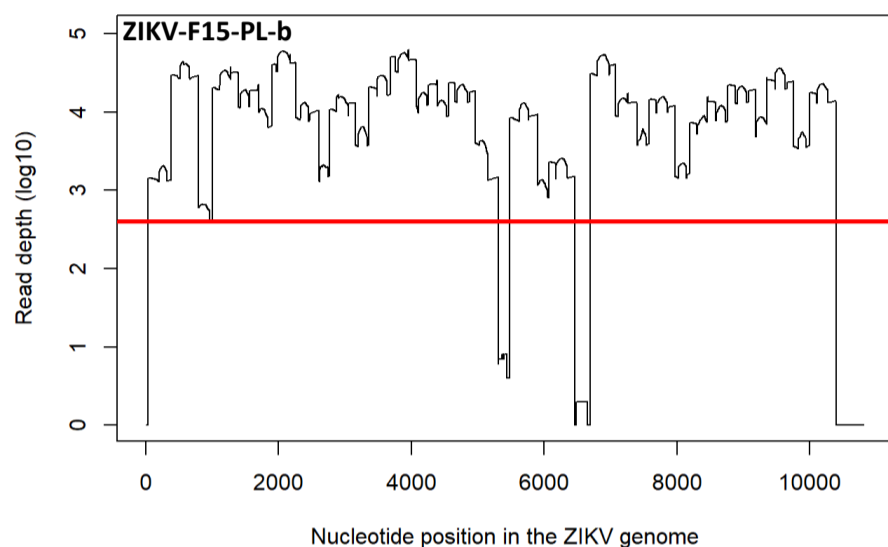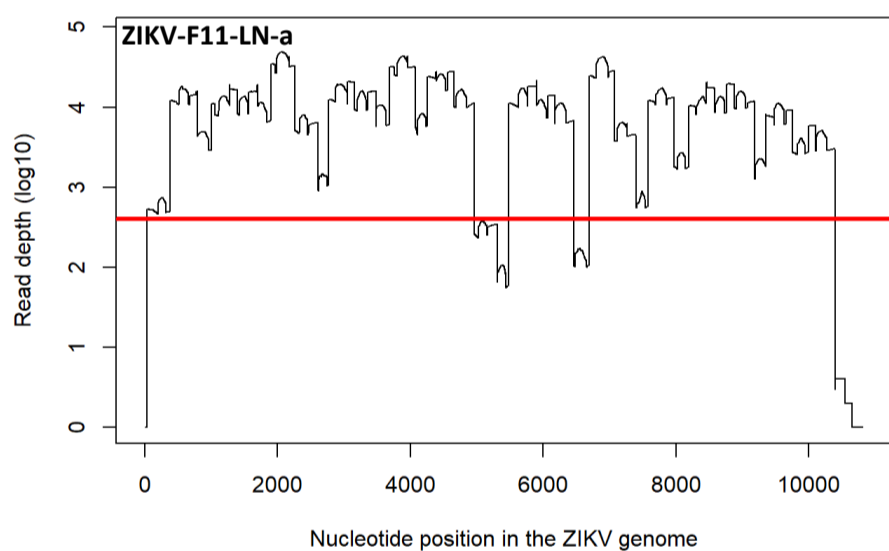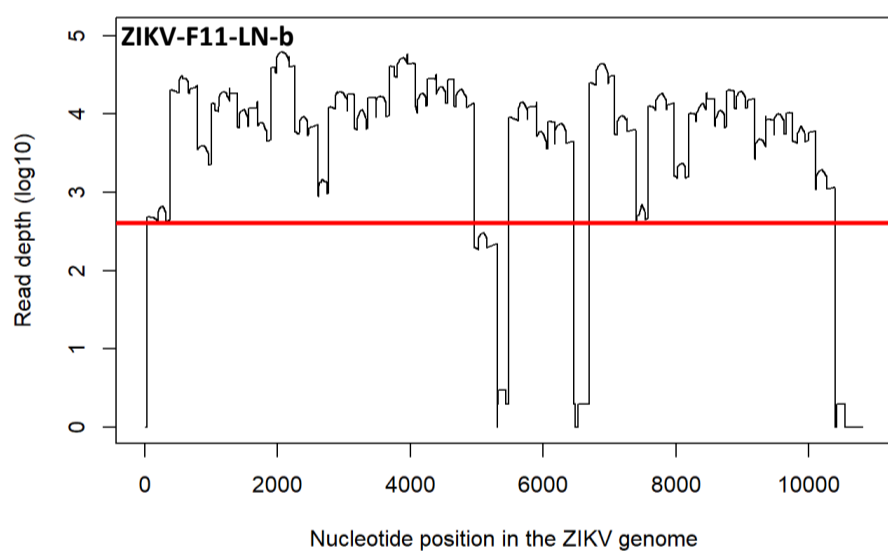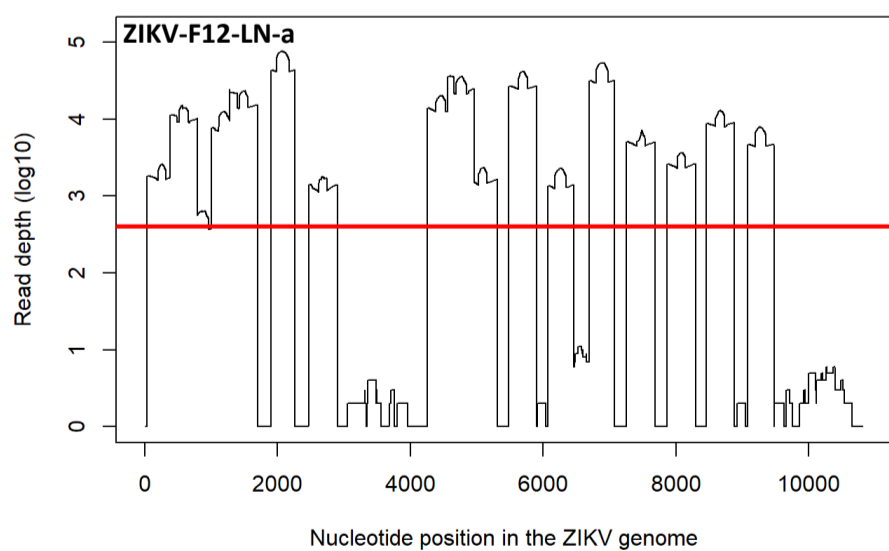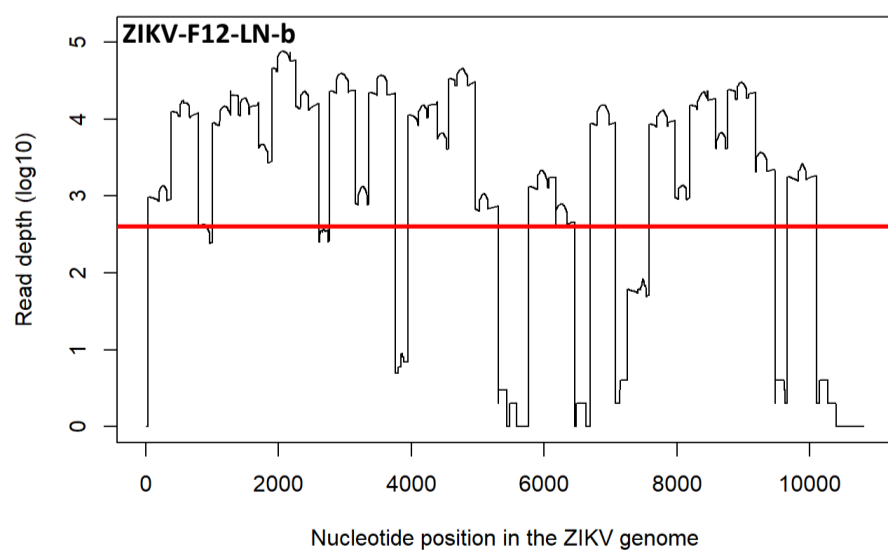

Supplement: Supplementary file 1 [file viruses-13-01827-s001.zip › Figure S1 Zika virus NGS coverage.pdf]

Figure S2. Porcine circovirus 2 NGS coverage

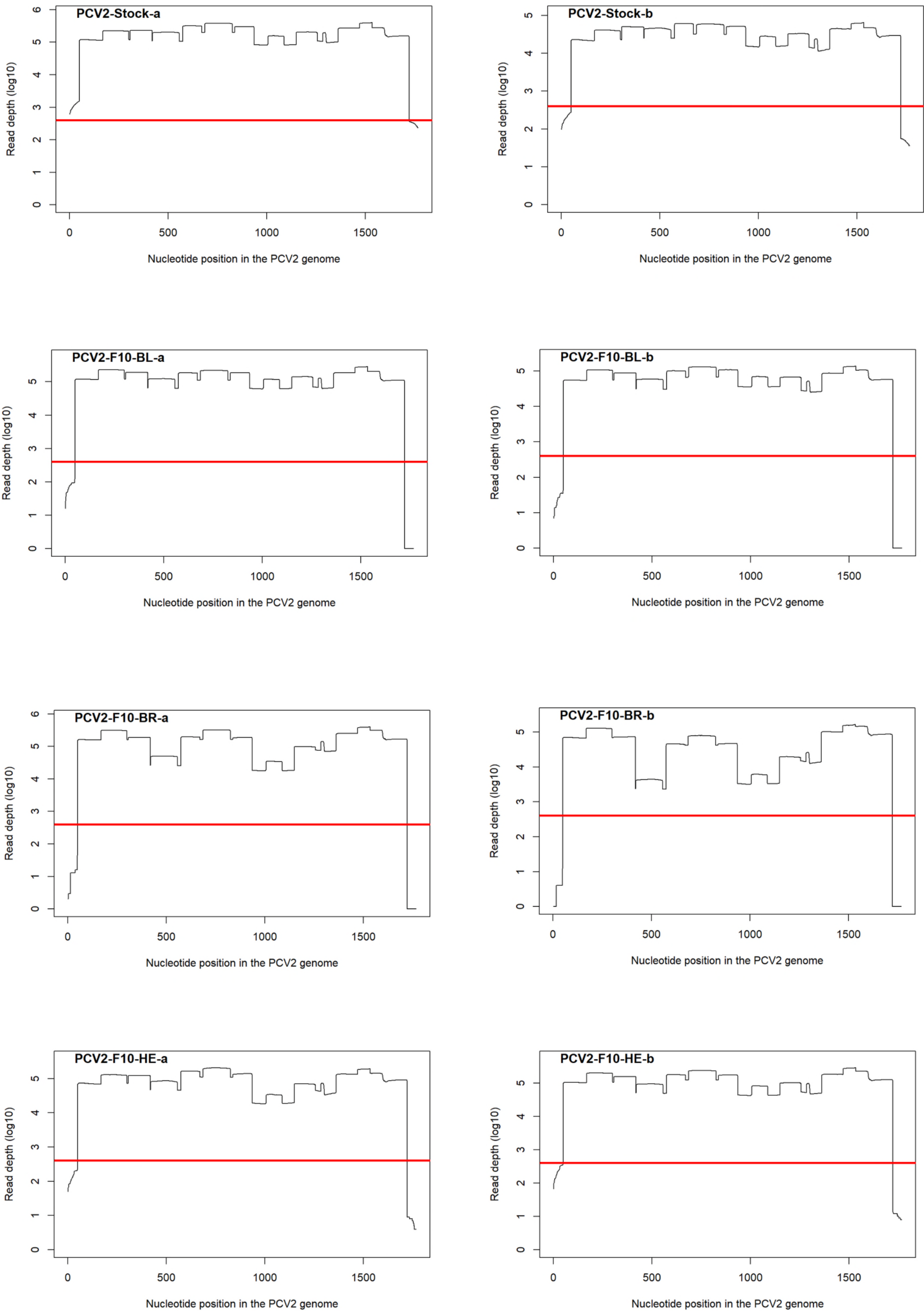

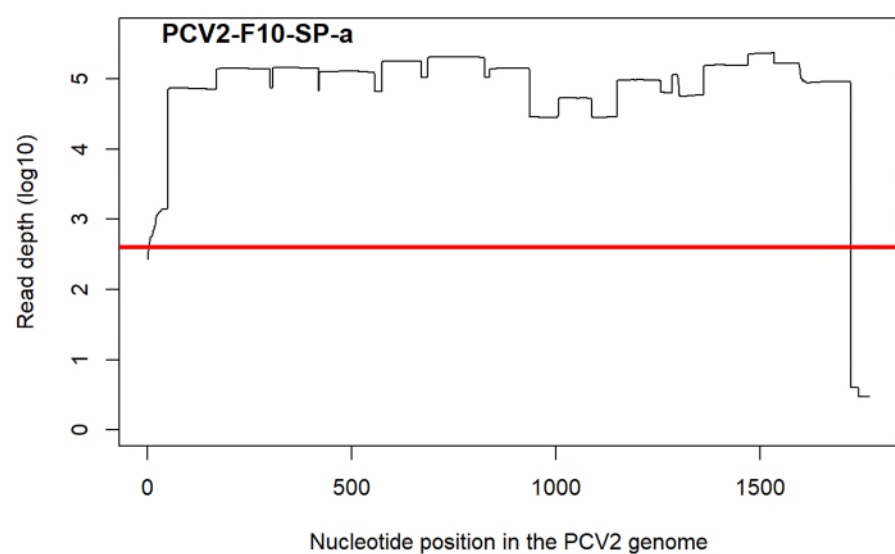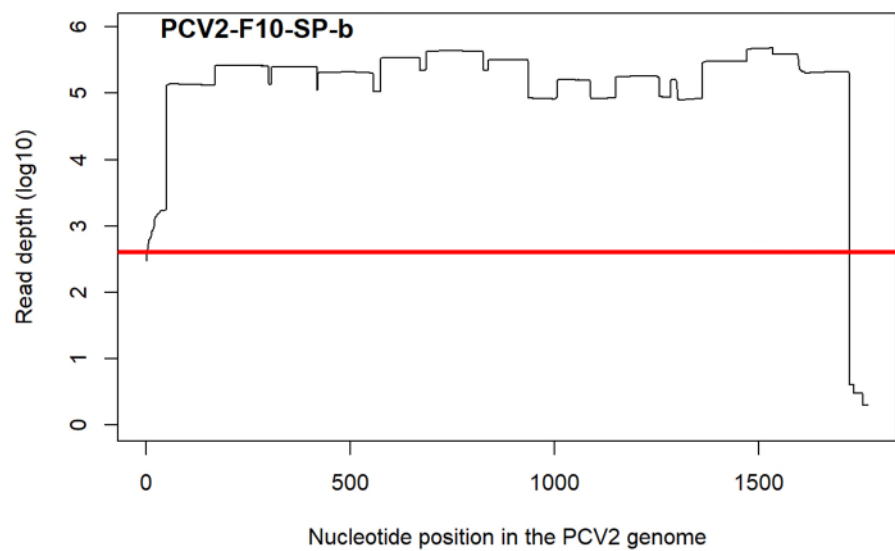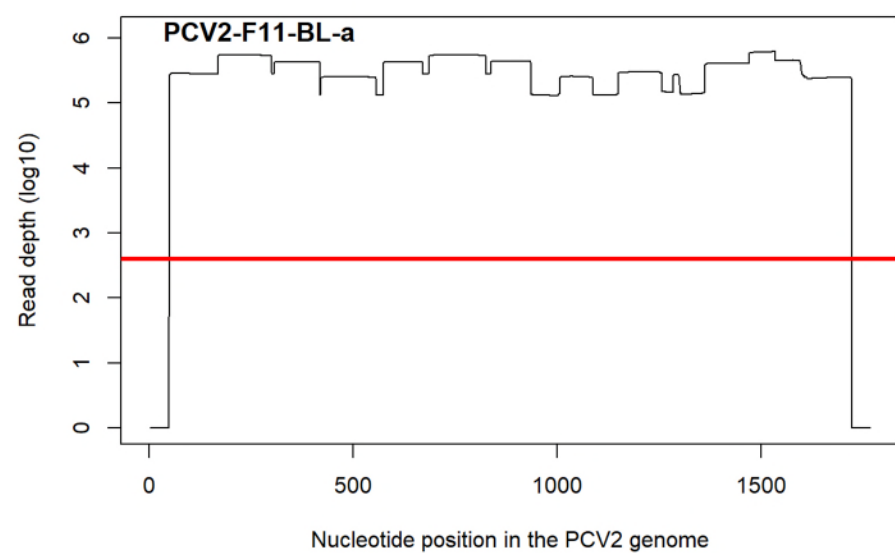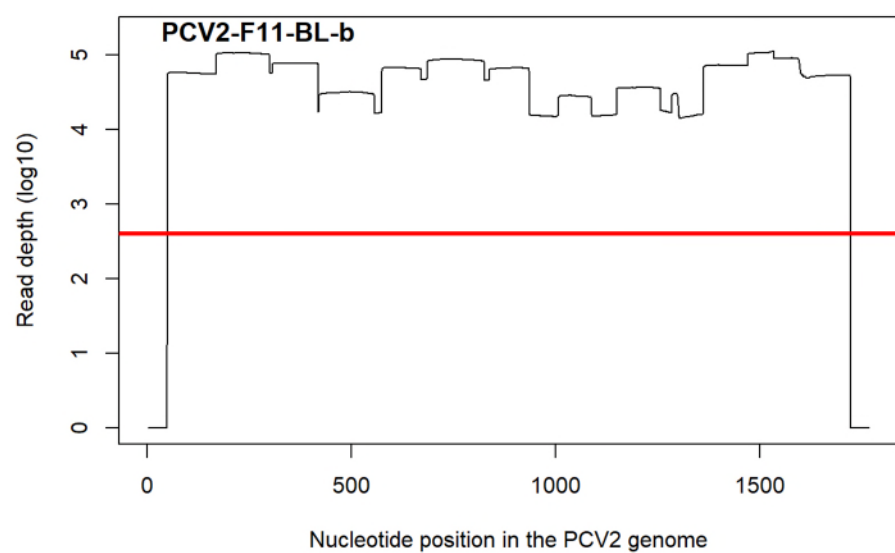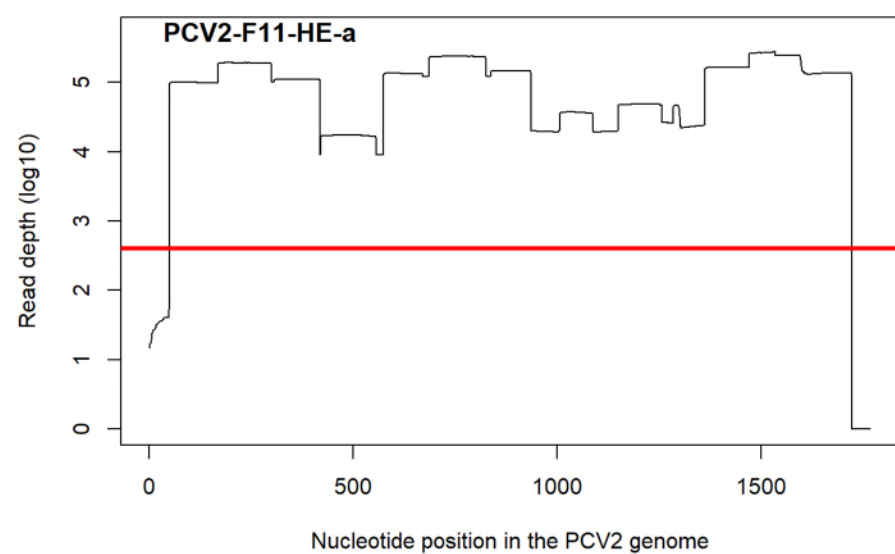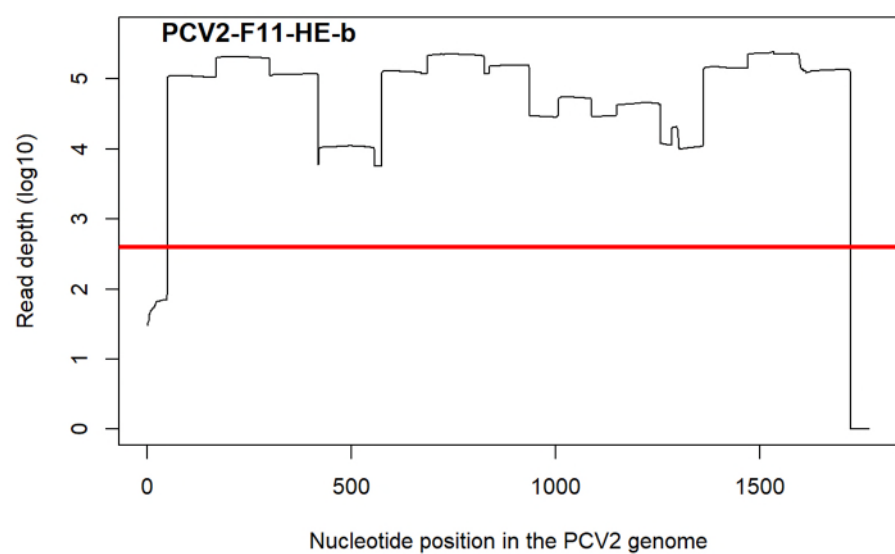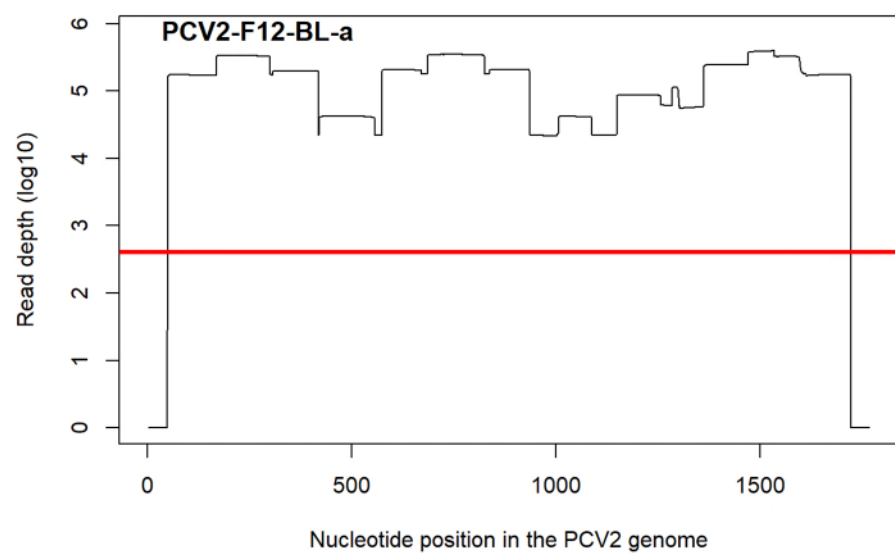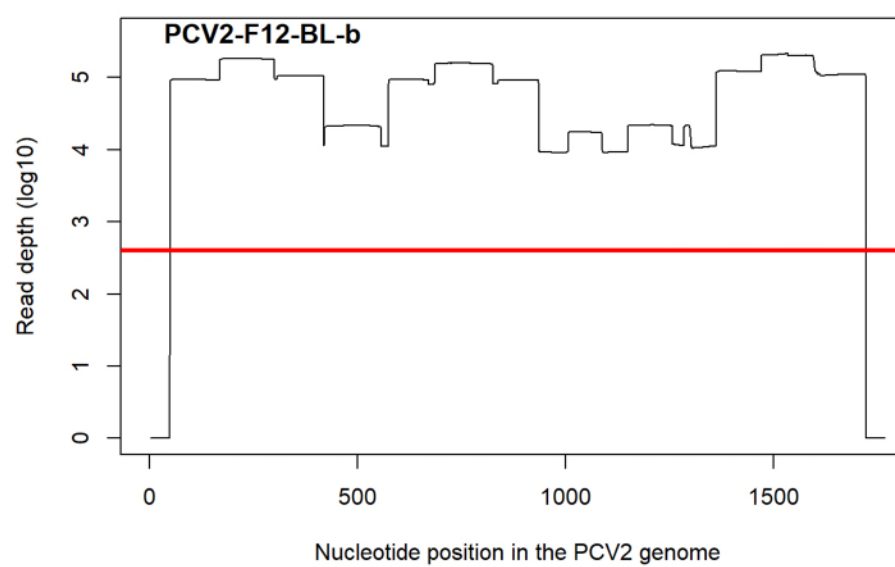

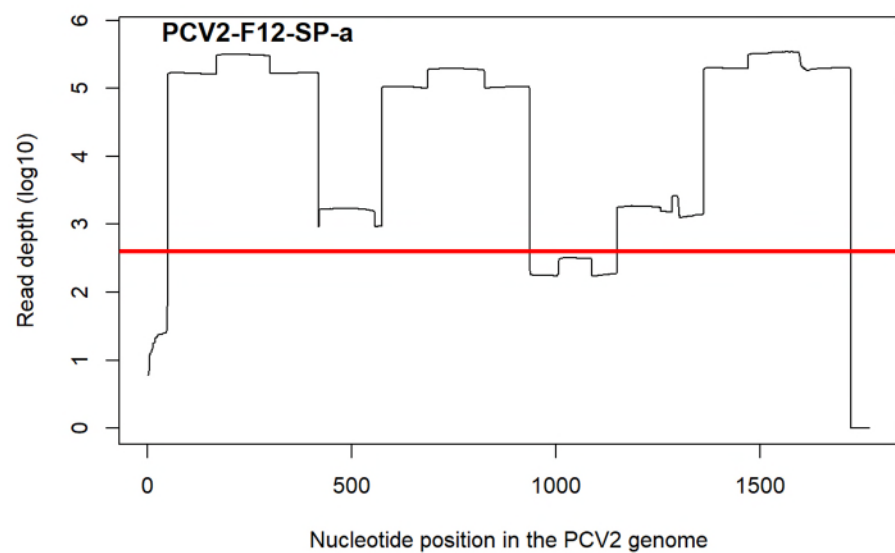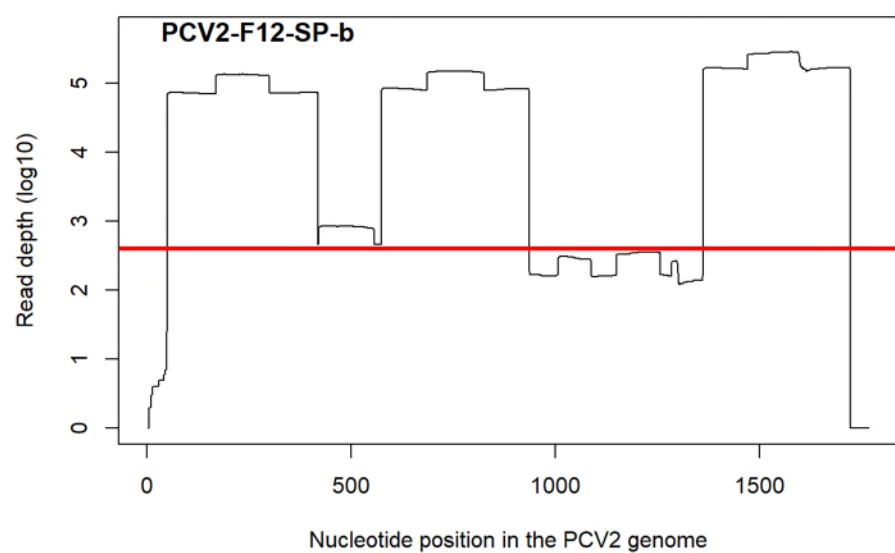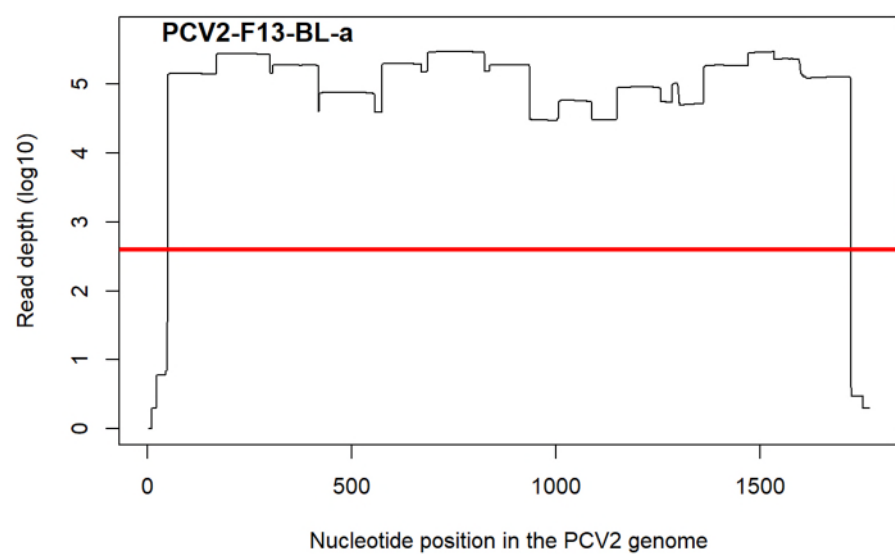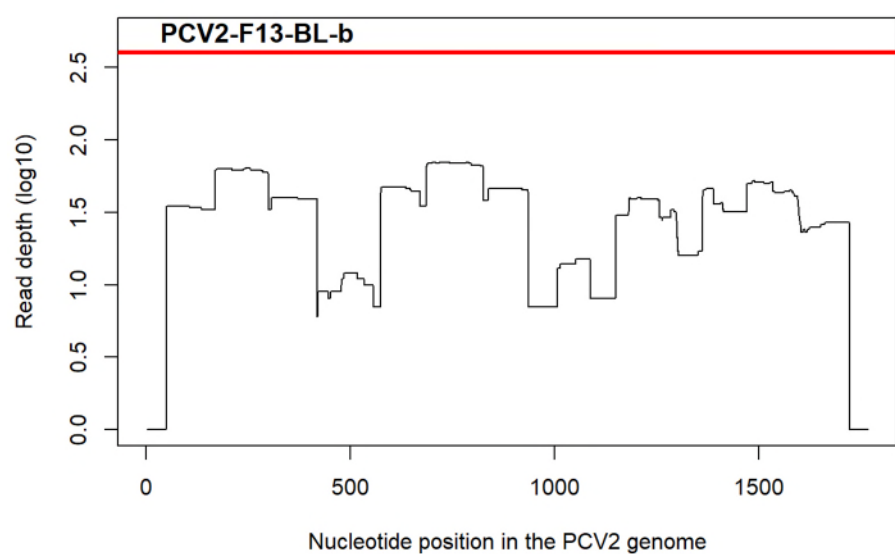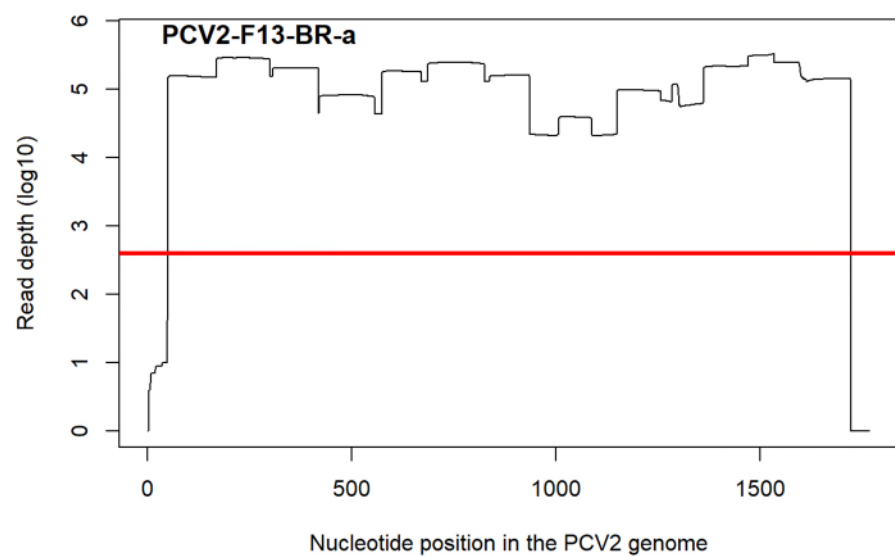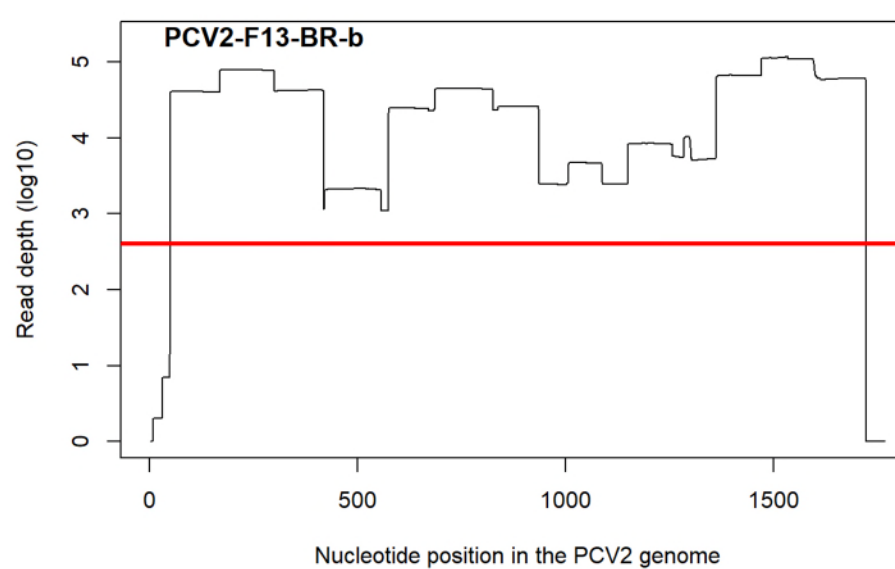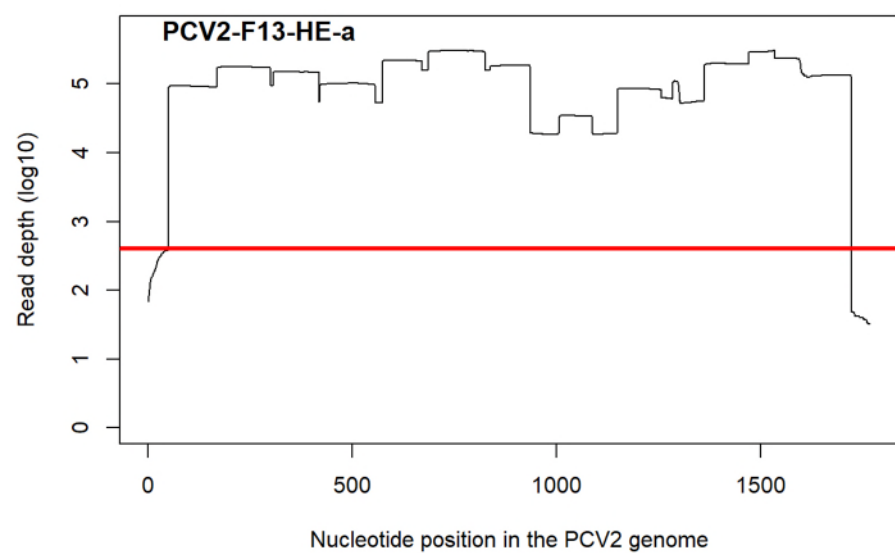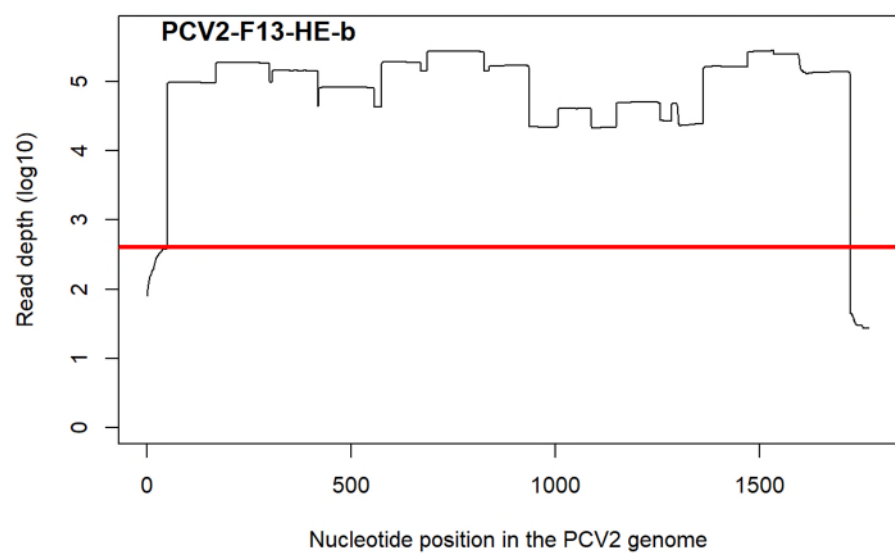

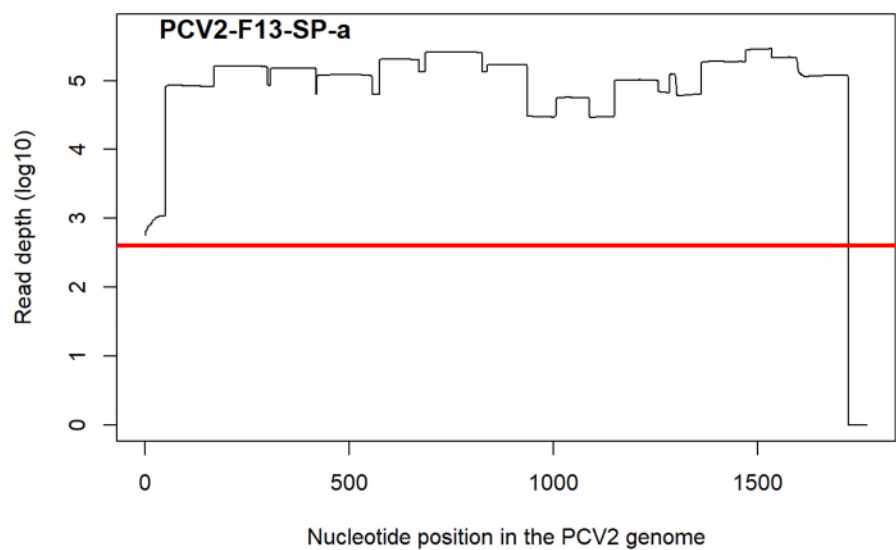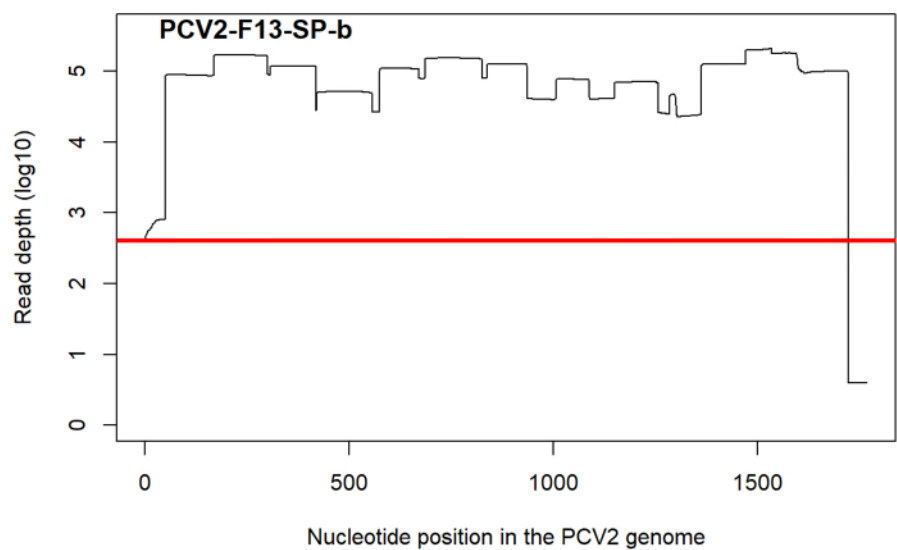

Supplement: Supplementary file 1 [file viruses-13-01827-s001.zip › Figure S2 Porcine circovirus 2 NGS coverage.pdf]
